# Bacterial nanocellulose production using Cantaloupe juice, statistical optimization and characterization

**DOI:** 10.1038/s41598-022-26642-9

**Published:** 2023-01-02

**Authors:** Noura El-Ahmady El-Naggar, A. B. Abeer Mohammed, Sahar E. El-Malkey

**Affiliations:** 1 Department of Bioprocess Development, Genetic Engineering and Biotechnology Research Institute, City of Scientific Research and Technological Applications (SRTA-City), New Borg El-Arab City, Alexandria, 21934 Egypt; 2 Microbial Biotechnology Department, Genetic Engineering and Biotechnology Research Institute, University of Sadat City, Sadat City, Egypt

**Keywords:** Biopolymers, Nanoparticles

## Abstract

The bacterial nanocellulose has been used in a wide range of biomedical applications including carriers for drug delivery, blood vessels, artificial skin and wound dressing. The total of ten morphologically different bacterial strains were screened for their potential to produce bacterial nanocellulose (BNC). Among these isolates, *Bacillus* sp*.* strain SEE-3 exhibited potent ability to produce the bacterial nanocellulose. The crystallinity, particle size and morphology of the purified biosynthesized nanocellulose were characterized. The cellulose nanofibers possess a negatively charged surface of − 14.7 mV. The SEM images of the bacterial nanocellulose confirms the formation of fiber-shaped particles with diameters of 20.12‒47.36 nm. The TEM images show needle-shaped particles with diameters of 30‒40 nm and lengths of 560‒1400 nm. X-ray diffraction show that the obtained bacterial nanocellulose has crystallinity degree value of 79.58%. FTIR spectra revealed the characteristic bands of the cellulose crystalline structure. The thermogravimetric analysis revealed high thermal stability. Optimization of the bacterial nanocellulose production was achieved using Plackett–Burman and face centered central composite designs. Using the desirability function, the optimum conditions for maximum bacterial nanocellulose production was determined theoretically and verified experimentally. Maximum BNC production (20.31 g/L) by *Bacillus* sp*.* strain SEE-3 was obtained using medium volume; 100 mL/250 mL conical flask, inoculum size; 5%, v/v, citric acid; 1.5 g/L, yeast extract; 5 g/L, temperature; 37 °C, Na_2_HPO_4_; 3 g/L, an initial pH level of 5, Cantaloupe juice concentration of 81.27 percent and peptone 11.22 g/L.

## Introduction

Cellulose is the most abundant natural polymer worldwide; it is an integral part of the primary cell wall of all plants, as well as many different kinds of algae and fungi. For industrial use, cellulose is obtained from wood pulp and cotton. Bacterial cellulose is environmentally benign nanostructured biopolymer produced mostly by a wide range of bacteria during microbial fermentation, the majority of which belong to the *Acetobacteraceae* family's *Gluconacetobacter* genus^1^. Genera include *Aerobacter*, *Agrobacterium*, *Rhizobium*, *Komagataeibacter* (formerly *Gluconacetobacter*), *Achromobacter*, *Sarcina*, *Enterobacter*, *Azotobacter*, *Pseudomonas*, *Alcaligenes*, *Escherichia*, *Salmonella*, *Burkholderia* and *Dickeya* can produce bacterial cellulose^2,3^. However, only *Gluconacetobacter* can efficiently produce more bacterial cellulose than other microbes^4^.

Plant cellulose and bacterial cellulose have the same chemical formula (C_6_H_10_O_5_)n. It is a homopolymer chain that is linear in shape and is composed of β-(1 → 4) glycoside linkages that connect glucose monomers^5^. As a result of the multiple hydrogen bonds that form between hydroxyl groups of glucose and oxygen molecules on the same or on a neighbour chain, it has a high tensile strength. Through the formation of internal and exterior hydrogen bonds, the glucose units and different bacterial cellulose fibrils interact tightly to produce a crystalline structure, resulting in the compacting of fibres that are completely water-insoluble but can be hydrated^6^. Compared with plant cellulose, the bacterial cellulose as a type of highly functional biopolymer has superior physicochemical and mechanical properties^7^. The bacterial cellulose pellicles have ribbon-shaped ultrafine nanofibers with < 100 nm^8^. The combination of the hydrophilic nature of the bacterial cellulose and the large surface area per unit of the thin nanofibers results in a greater water-absorbing and retention capacity, improved adherence, and increased moisture content^9^. It possesses an array of unique properties, including a high degree of polymerization, a higher crystallinity, good biocompatibility, high elasticity, mould resistant, a higher tensile strength properties, durability, renewability, a high degree of biodegradability, nontoxic and non allergenic features, easy to isolate and purify due to the fact that it is produced free of hemicelluloses, pectins and lignin and relatively simple low-cost production^10–13^.

The higher-order bacterial cellulose are formedduring fermentation and then aggregate to a ribbon-shaped microfibrils, which is produced with a width of less than 100 nm^14^ and belong to the thinnest naturally occurring fibers. Moreover, the microfibrils of bacterial cellulose are crystallized into bundles, and these bundles are then stacked on top of each other to create ribbons^15^ that trap the bacterial cells. It is presumed that the ribbons serve to protect the bacteria from dehydration and ultraviolet (UV) light. Additionally, the ribbons makes it possible for bacteria to become floatable by entrapping the carbon dioxide that is produced by the tricarboxylic acid cycle. Since, these bacteria are obligate aerobes, the cellulose biofilm is a way to keep the bacteria attached so that they can reach the air–liquid interface where there is oxygen availability^16^.

The bacterial cellulose has great potential as a material in a variety of new applications in the food industry, papermaking, high-end acoustic diaphragms, cosmetics, biomedicine and other fields^17^. Additionally, Hu et al.^18^ stated that the functional nanomaterials derived from bacterial nanocellulose are being used as optoelectronic materials and devices, photocatalytic nanomaterials, sensors, and magnetically responsive membranes. The most exciting applications of the bacterial cellulose have already been documented in the biomedical field including dental implants, medical pads^11^ cartilage scaffolds^19^ vascular grafts, artificial blood vessels for microsurgery^20^, and artificial skin designed for use on humans suffering from severe burns^21^ as the bacterial cellulose is non-allergenic, has a high hydrophlicity, and can be disinfected without losing its properties. Carvalho et al.^22^ demonstrated that the numerous advantages of bacterial nanocellulose, including its biocompatibility, ability to absorb exudates during the inflammatory reaction, ability to maintain a hydration within wounds, conformability, elasticity and transparency, make it a promising candidate for use in wound therapy approaches. The use of bacterial nanocellulose has been shown to have a significant analgesic effect, as well as to hasten the process of new epithelial tissue generation and the formation of granulation tissue, and to reduce the formation of scar tissue^23,24^. Fu et al.^25^ demonstrated that the bacterial nanocellulose can act as a natural framework for the healing of a diverse array of tissues due to its unique nanoscaled three-dimensional network structure.

The bacterial cellulose has limited antioxidant ability against reactive oxygen species^26^. Heavy metals could be removed with the use of the bacterial cellulose as an adsorbent^27^. When compared to other celluloses, the bacterial cellulose has more surface hydroxyl and ether groups, which can act as active sites for improved metal ion adsorption^10^. A potential application for fragmented bacterial nanocellulose is in the papermaking industry, enabling for the manufacturing of flexible/durable papers as well as papers with a high filler content, which are great for use as banknote paper^28^. Mautner et al.^29^ proved that BNC-based nanopaper was appropriate for ultrafiltration operations requiring high filtration efficiency. Pure cellulose can be employed as a thickening and stabilising factor in processed foods, as well as to promote gelling and water binding. Additionally, cellulose-producing bacteria can be cultivated in culture media containing fruit syrup, which allows the cellulose generated to absorb the fruit's natural flavour and pigment. Due to the fact that bacterial nanocellulose can be made in a variety of shapes and textures, it has numerous applications in food as a sort of dietary fibre^30^. Müller et al.^31^ found that the bacterial nanocellulose can be used in a protein drug delivery system with serum albumin as a model drug.

Although the bacterial cellulose has great potential as a material in a variety of new applications, its low productivity and production cost prevent it from being mass-produced on a large scale. The most significant factor affecting the total cost of the bacterial cellulose production is the culture medium. Numerous studies have been conducted over the last decade to optimize the bacterial nanocellulose production, including pH regulation, the use of alternative carbon sources like sucrose, molasses, sugar cane and rotten fruit, as well as culture in both a static and agitated environment.

The production of bacterial cellulose commonly takes place in the HS medium, which uses glucose as its principal source of carbon and includes peptone and yeast extract as its sources of nitrogen. However, using glucose as a carbon source for the production of bacterial cellulose is not only quite expensive, but it also results in the formation of gluconic acid as a by-product, which lowers the pH of the culture and, as a consequence, decreases the amount of bacterial cellulose that is produced^32^. Thus, one challenging and crucial aspect of the fermentation process and the bacterial cellulose production is identifying low-cost approaches and culture media that can significantly improve the bacterial cellulose production in a short period of time^33^. As a result, researchers have investigated the agro-industrial by-products, non-traditional and less expensive carbon and/or nitrogen sources for the bacterial cellulose production as interesting alternatives. In addition to the most commonly used carbon sources (glucose and sucrose), alternative carbohydrates such as galactose, xylose, mannose, fructose and glycerol have been studied effectively^34^.

The objectives of this study were to isolate a novel bacterial source capable of producing bacterial cellulose with a high capacity from a low-cost carbohydrate source, thereby reducing the production cost, to improve the efficiency of the bacterial cellulose production process for commercial and industrial use, and to investigate the purity, structure, and properties of the obtained bacterial nanocellulose.

## Materials and methods

### Isolation of *Bacillus* spp.

The bacterial isolates were isolated from various soil samples collected from different places of the residential area; Barhiem, Menoufia governorate, Egypt. The bacterial isolates had been isolated using standard dilution plate method procedure on Petri plates containing agar medium of the following composition: 0.5 g of Locust bean gum; 6.78 g Na_2_HPO_4_; 3.0 g KH_2_PO_4_; 1.0 g NH_4_Cl; 0.5 g yeast extract; 0.5 g NaCl; 1 mL of 1 M CaCl_2_; 1 mL of 1 M MgSO_4_; 20 g agar and distilled water up to 1L. Nystatin (50 μg/mL) was incorporated as an antifungal agent to minimize fungal contamination. Petri plates of the previous medium were inoculated with a loopful of soil suspension, then incubated for 24 h at 30 °C. The inoculated plates were examined for the appearance of bacterial colonies. The bacterial colonies that exhibited culture features typical of *Bacillus* species, such as thick and opaque; cream-colored, round or irregular were subcultured and purified on nutrient agar plates. These strains were stored as spore suspensions in 20% (v/v) glycerol at − 20 °C for subsequent investigation. The purified *Bacillus* species were then screened for their ability for nanocellulose production to find a better producer of bacterial cellulose before being identified.

### Inoculum preparation

In order to prepare the inoculum, the bacterial cells were cultivated in 250 mL Erlenmeyer conical flasks containing 100 mL of the medium containing (g/L): glucose (20), yeast extract (5), peptone (5), Na_2_HPO_4_ (2.7) and citric acid (1.15), pH was adjusted to 5. The medium was autoclaved for 20 min at 121 °C. The bacterial cells were grown under static conditions for 24 h at 30 °C, this was considered the standard inoculum for the present investigation.

### Screening of different bacterial isolates potentialities for production of bacterial nanocellulose

Two media were used for screening the bacterial isolate for BNC production under static-flask culturing. The first medium contains (g/L): yeast extract (5), peptone (5), glucose (20), citric acid (1.5), and Na_2_HPO_4_ (2.7), and distilled water up to 1L; the initial pH was adjusted to 5 using NaOH (1 M)^35,36^. The second medium contains (g/L): yeast extract (5), peptone (5), glucose (20), Na_2_HPO_4_ (2.67) and citric acid until pH 3.6^37^. An inoculum of 10% (v/v) from stock culture was used to inoculate the two-broth media, then incubated under static conditions for 14 days at 30 °C.

### Harvesting, purification and quantification of the bacterial nanocellulose

Following the fermentation, the membranes of bacterial nanocellulose (BNC) layers, which are synthesized and secreted in contact with the air as the exopolysaccharides were harvested through picking up, purified and quantified. BNC was washed three times with distilled water, boiled in a distilled water at 70 °C for 3 h^38^, then soaked in 0.1 M NaOH solution for 3 h at 80 °C to remove the medium components and dissolve the bacteria cells possibly entrapped in the nanocellulose microfibers.

Heating with NaOH improves viscosity, removes specific metabolites, hence promoting purification and the removes cellulose with the low molecular weights, which results in a biomaterial with improved properties^39^. After the bacterial nanocellulose turned transparent, the granules were washed thoroughly with distilled water to neutralize them (complete alkali removal). The purified bacterial nanocellulose was dried at 50 °C until it reached a constant weight^40^. The nanocellulose yield was expressed as gram dry mass per Liter.

### Evaluation of various carbon sources for the production of bacterial nanocellulose by a selected strain

The influence of various carbon sources on the production of bacterial nanocellulose by a selected strain was evaluated on the previously mentioned two culture media. The fermentation was performed in liquid culture media under static conditions Using Erlenmeyer flasks with 250-mL capacity, each containing 100 mL of culture medium. Ten carbon sources (at 2 percent; glucose, glycine, mannitol, fructose, starch, ribose, xylose, sucrose), Cantaloupe juice and *Ulva lactuca* biomass extract (%, v/v), were sterilized and added to the sterilized medium to determine a more appropriate source of carbon to produce the bacterial nanocellulose up to 14 days. The amount of the bacterial nanocellulose produced (in g/L dry mass) was determined.

*Ulva lactuca* biomass was collected and extensively rinsed with seawater to eliminate any contaminants, adherent sand particles, or epiphytes. Under ambient temperature, Seaweed was thoroughly washed under running tap water to eliminate salts and then dried to remove moisture. The extraction method according to the modified procedures described by Latique et al.^41^. In a 250 mL flask, 20 g of the dried crushed algal biomass was mixed with 100 mL of distilled water and boiled separately for one hour in water bath, then the mixture was filtered to remove debris. This filtrate represented a 100% algal crude extract.

The Cantaloupe fruits (*Cucumis melo*) have been processed into clarified juice by squeezing the frozen and thawed Cantaloupe flesh in a blender with the peel removed and then filtered. The sugar composition values of Cantaloupe juice were (g/100 mL): sucrose 1.73, glucose 1.23 and fructose 1.61^42^.

### Identification of the most promising bacterial isolate (strain SEE-3)

The isolated bacteria were screened for BNC production, the most talented isolate was selected for characterization through investigating the morphology (culture and cell), Gram staining, and spore formation. The biochemical tests, including carbon utilization, enzymatic activities, inhibition and resistance, were also carried out with aid of VITEK 2 systems. The bacterial cells were also investigated using the scanning electron microscopy (SEM).

The molecular-based identification was performed, using 16S rRNA sequencing. Thermo Gene JET Genomic DNA Purification Kit (#K0721) was used to extract the bacterial genomic DNA. The 16S rRNA gene was amplified by PCR following the protocol of El-Naggar et al.^43^. The Qiaquick spin-gel extraction kit (Qiagen) was used in order to purify the product of the PCR. The universal primers: 1492R reverse primer (5′‐TACGGYTACCTTGTTACGACTT‐3′) and 27F forward primer (5′-AGAGTTTGATCCTGGCTCAG-3′) were used. The acquired 16S rRNA gene sequence was matched to the publicly available 16S rRNA gene reference sequences in the GenBank databases using the BLASTN^44^. MEGA version X software (https://www.megasoftware.net/) was used to construct the phylogenetic tree^45^.

### Solubility of BNC in water and standard organic solvents

The solubility of BNC produced by *Bacillus* sp*.* strain SEE-3 was investigated in water, standard organic solvents (ethanol, chloroform, dimethyl sulfoxide (DMSO), propanol, xylene, methanol, butanol, isopropanol, acetic acid), ammonia solution and a mixture of 7% NaOH, 12% urea, and 81% distilled water^46^.

### SEM and TEM investigation of BNC samples

The size, morphology, and structure of the bacterial nanocellulose samples produced by *Bacillus* sp*.* strain SEE-3 coated with gold using a sputter coater (SPI-Module) and were analysed by a scanning electron microscope (SEM) “JSM-5500 LV; JEOL, Ltd- Japan; by using high vaccum mode operating at 15 kV at the Regional Center of Mycology and Biotechnology, Al-Azhar University, Cairo, Egypt”. The samples were also examined with SEM “model JEOL-JSM-IT200; at 20 kV at the Electron Microscope Unit, Faculty of science, Alexandria University, Alexandria, Egypt”. The samples were examined with a Transmission Electron Microscope (TEM) “JEM-2100 Plus, JEOL Ltd., Japan; at the Central Laboratory, City of Scientific Research and Technological Applications, Alexandria, Egypt”.

### Influence of ultrasounds on morphology of bacterial cellulose nanofibers

Sample of the bacterial nanocellulose produced by *Bacillus* sp*.* strain SEE-3 was suspended in 1 mL of 99.5% ethanol as volatile solvent and the resulting suspension was sonicated for 10 min in an ultrasonic bath Branson (Co., Shelton, USA (50/60 Hz, 125 W) “model B*-*220 SmithKline”*.*

### Thermogravimetric analysis (TGA) and Differential scanning calorimetry (DSC)

The glass transition (T*g*) and melting temperature (T*m*) were measured in order to determine the thermal behaviour of the bacterial nanocellulose. The DSC and TGA analyses of the bacterial nanocellulose were performed to investigate its thermal behaviour. TGA was performed using TGA-50H Thermogravimetric analyzer on bacterial nanocellulose sample of about 6 mg. The sample was scanned at a flow rate of 40 mL/min over a temperature ranging from room temperature to 800 °C. The thermal behavior of the bacterial nanocellulose sample was estimated using DSC. DSC technique is used to determine how a material responds to changes in temperature or time. The thermogram behavior was explored up to 400 °C.

### Fourier transforms infrared spectroscopy (FTIR) analysis

In order to examine the surface properties of the bacterial nanocellulose in comparison with avicel, FTIR spectroscopy analysis was carried out. For surface properties investigation, the BNC samples used for FTIR measurements were ground with KBr Pellets. The Shimadzu FTIR-8400 S spectrophotometer was used to measure the FTIR spectra at a resolution of 1 cm^−1^ in the range of 4500–500 cm^−1^.

### Zeta potential analysis

The zeta potential (ζ) was measured at “central laboratories, City of Scientific Research and Technological Applications, Alexandria, Egypt” using a Malvern 3000 Zetasizer Nano ZS, UK” to determine the surface charge properties of the bacterial nanocellulose sample. The bacterial nanocellulose suspension was diluted to a 0.01 wt% concentration with deionized water. Prior to the test, the diluted solution was homogenised in a high-speed homogenizer at a speed of 13,000 rpm for 10 min and then maintained in an ultrasonic bath. The sample was analyzed three times. The measurements were performed at 25 °C.

### X-ray diffraction (XRD)

XRD was employed to evaluate the pattern and crystallinity degree of the bacterial nanocellulose. At ambient temperature, the X-ray diffraction patterns were recorded Ni-filtered Cu Kα radiation (λ = 1.54 A°). Diffractmeter Type: Bruker D2 Phaser 2nd Gen. The generator current (mA) and operating voltage (kV) were 30 and 10; respectively. Data were collected at a rate of two degrees per minute between 5 and 60 degrees 2θ. The degree of crystallinity of bacterial nanocellulose sample was determined using the empirical method proposed by Segal et al.^47^ equation from the diffracted intensity data:

1 $${\text{CrI}}^{{{\text{XRD}}}} (\% ) = \frac{{I_{002} - I_{{{\text{am}}}} }}{{I_{002} }} \times 100$$

I_002_ is the intensity value for the crystalline cellulose, and I_am_ is the intensity value for the amorphous cellulose.

### Selection of significant variables using Plackett–Burman design (PBD)

Plackett–Burman (PBD)^48^ is a two-factorial design, it is very useful for screening the most significant physicochemical factors that are required for an increased response with respect to their main effects^49^. Therfore, PBD was used in the current study to define the significant process physico-chemical factors that influence the production of bacterial nanocellulose by *Bacillus* sp*.* strain SEE-3. The influence of ten nutritional and environmental factors were evaluated for their effects on the bacterial nanocellulose production using a Plackett–Burman experimental design including: A (medium volume; mL/250 mL conical flask), B (pH), C (incubation time; days), D (inoculum size; %, v/v), E (Cantaloupe juice; %, v/v), F (citric acid; g/L); G (peptone; g/L), H (yeast extract; g/L), J (temperature; °C), K (Na_2_HPO_4_; g/L) in addition to one dummy variable. 12-run Plackett–Burman experimental design matrix was used to screen for significant factors influencing bacterial nanocellulose synthesis by *Bacillus* sp*.* strain SEE-3 under static fermentation. The lower and higher levels of the parameters are based on our preliminary research. The experimental design of Plackett–Burman relies on the following polynomial equation of the first order:

2 $${\varvec{Y}} = {\varvec{\beta}}_{{\mathbf{0}}} + \sum {{\varvec{\beta}}_{{\varvec{i}}} {\varvec{X}}_{{\varvec{i}}} }$$

where Y is the bacterial nanocellulose production, *β*_0_ is the intercept for the model and *β*_*i*_ is the linear coefficient, while *X*_*i*_ is the level of the independent variables.

The Plackett–Burman design does not define the mutual interactions between the process variables; rather, it is employed to screen for and identify significant variables that influence the response^50^. As a result, the face-centered central composite design (FCCCD) was employed to define the levels of significant variables and to investigate the interaction effects among multiple significant variables.

### Face centered central composite design (FCCCD)

FCCCD is an efficient design that is widely used in optimization processes because it provides a sufficient amount of information for validating accuracy of the model without requiring a large number of experimental runs, thereby lowering the overall cost of the experiment^51^. Based on Plackett–Burman experiment results, FCCCD was used to investigate and optimize the levels and to study the interaction effects among the most significant independent variables that affect the bacterial nanocellulose production. The most significant three variables (pH, peptone and Cantaloupe juice) were selected and studied at three different levels which were the low (− 1), centre (0), and high (1) levels. The zero levels (central values) chosen for the experiments were: pH 5, peptone (10 g/L) and Cantaloupe juice (75%, v/v). A total of 20 experiments were performed in order to optimize the levels and to study the interaction effects among the chosen factors on the bacterial nanocellulose synthesis by *Bacillus* sp*.* strain SEE-3. Twenty runs were conducted in a 250 mL Erlenmeyer flask containing 100 mL of media prepared according to the design. After the media had been inoculated, they were incubated at 37 °C. In order to fit the obtained experimental data of FCCCD, the following polynomial equation of the second order was applied:

3 $${\varvec{Y}} = {\varvec{\beta}}_{{\mathbf{0}}} + \sum\limits_{{\varvec{i}}} {{\varvec{\beta}}_{{\varvec{i}}} {\varvec{X}}_{{\varvec{i}}} } + \sum\limits_{{{\varvec{ii}}}} {{\varvec{\beta}}_{{{\varvec{ii}}}} {\varvec{X}}_{{\varvec{i}}}^{2} } + \sum\limits_{{{\varvec{ij}}}} {{\varvec{\beta}}_{{{\varvec{ij}}}} {\varvec{X}}_{{\varvec{i}}} {\varvec{X}}_{{\varvec{j}}} }$$

where* Y* is the predicted bacterial nanocellulose production, X_i_ is the coded levels of independent factors. The β_0_, β_i_, β_ii_, β_ij_ denotes the regression, linear, quadratic and β_ij_ the interaction coefficients; respectively.

The experiments were repeated twice, and the average of the obtained bacterial nanocellulose produced by *Bacillus* sp*.* strain SEE-3 used as the response.

### Statistical analysis

Using the Windows edition of Design-Expert software (version 12, Stat-Ease, Minneapolis, USA) (https://www.statease.com/software/design-expert/), the experimental design and statistical analysis were both carried out. For the purpose of drawing three-dimensional and contour surface plots, the STATISTICA version 8 programme was applied (https://www.statsoft.de/de/software/statistica).

## Results and discussion

The total of ten distinguishable bacterial strains (coded SEE-1 to SEE-10) were evaluated for their ability to produce bacterial nanocellulose. Among these isolates, 4 isolates were found positive for nanocellulose production. The four strains that produced bacterial nanocellulose were SEE-3, SEE-7, SEE-9, and SEE-10. Based on the dry weight of the bacterial nanocellulose produced (g/L of medium), strains SEE-3, SEE-9, SEE-10 and SEE-7 produced 9.4, 6.2, 5.9, and 3.7 g/L, respectively (Supplementary Table S1). *Bacillus* sp. strain SEE-3 was found to have more potential than the other strains, as it displayed the largest production of bacterial nanocellulose (9.4 g/L of medium), and it could a promising candidate for BC production. Therefore, it was selected for further nanocellulose production and studies.

### Effect of various carbon sources on the production of bacterial nanocellulose

The low productivity of the bacterial nanocellulose is one of the industry's application obstacles. Nowadays, numerous carbon sources, including oligosaccharides, organic acids, monosaccharides and alcohol have been used to enhance the bacterial nanocellulose biosynthesis^38,52^. The influence of various carbon sources and a low-cost carbon substrates on the bacterial nanocellulose production by *Bacillus* sp*.* strain SEE-3 have been evaluated for the bacterial nanocellulose production on two different media.

**Figure 1:**
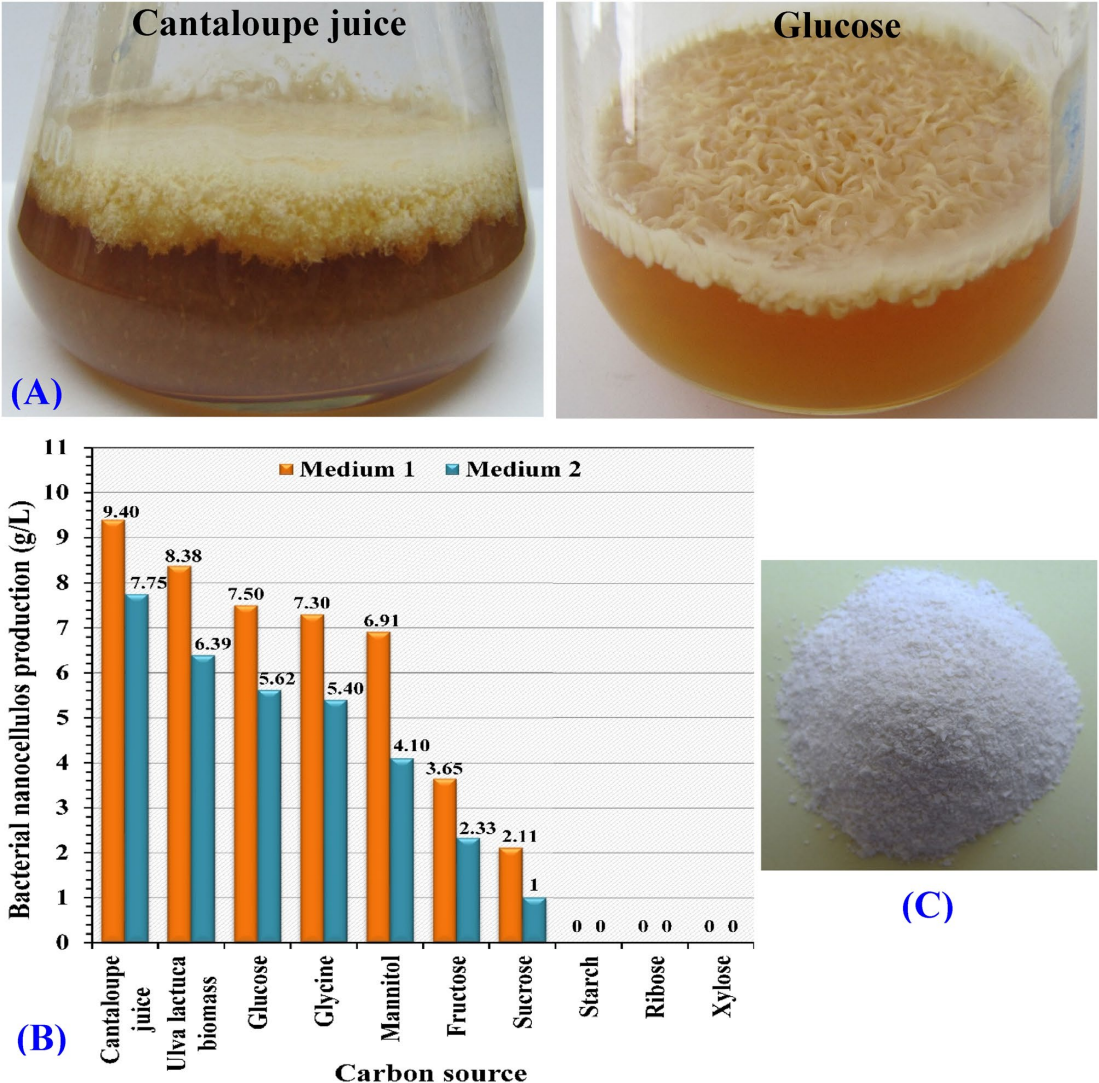
(**A**) Bacterial cellulose layer produced at the air–liquid interphase. (**B**) Purified powder of the bacterial nanocellulose. (**C**) Effect of ten carbon sources on bacterial cellulose production after 14 days of static cultivation on different carbon sources at 37 °C.

After inoculation of the medium 1 and medium 2, the bacterial nanocellulose layers were produced by *Bacillus* sp*.* strain SEE-3 through static fermentation at 30 °C for 7–14 days. As shown in Fig. 1A, the bacterial nanocellulose was observed on the culture medium surface as layer and the dry weight of the produced bacterial nanocellulose was quantified after the fermentation and purification processes.

A clear difference was observed for the bacterial nanocellulose production from different carbon sources in the culture medium 1 and 2. *Bacillus* sp*.* strain SEE-3 has a strong ability to produce the bacterial nanocellulose using Cantaloupe juice followed by *Ulva lactuca* biomass extract (Fig. 1B). On the basis of dry weight of the bacterial nanocellulose production (g/L medium), the highest bacterial nanocellulose production was obtained on in the culture medium no.1. The bacterial nanocellulose production was 9.4, 8.38, 7.5, 7.3, 6.91, 3.65 and 2.11 g/L by using Cantaloupe juice (100%, v/v) followed by *Ulva lactuca* biomass extract (100%, v/v), at 2% w/v carbon source concentration of glucose, glycine, mannitol, fructose and sucrose; respectively (Fig. 1B). The bacterial nanocellulose was not produced on xylose, ribose and starch by *Bacillus* sp*.* strain SEE-3.

Our findings are consistent with those of Castro et al.^53^, Embuscado et al.^54^ and others who have found that the type of carbon sources influence the formation of bacterial cellulose production in bacteria. Glucose, sucrose, and fructose have all been identified as suitable carbon sources for production of bacterial nanocellulose. The effect that carbon sources have on the growth of microorganisms and the production of metabolites is affected by a number of parameters, one of which is the concentration of carbon. Previous research by Ramana et al.^55^ demonstrated that the maximum production of bacterial nanocellulose by *Acetobacter xylinum* was found by using a variety of carbon sources, which include glucose, sucrose, mannitol and fructose. Sucrose was found to be a more effective carbon source. Molina-Ramírez et al.^37^ found that the maximum yields of bacterial cellulose production by *Komagataeibacter Medellinensis* were 2.80, 0.38, and 1.68 g/L when glucose, fructose, and sucrose were supplied at a concentration of 2% w/v; respectively. Mohammadkazemi et al.^56^ and Kim et al.^57^ reported that sucrose had the highest levels of bacterial cellulose biosynthesis as a carbon source which was greater than that achieved by utilizing fructose. However, Embuscado et al.^54^ claimed that the production yield of bacterial cellulose in fructose-based medium is greater than that of sucrose. Castro et al.^53^ and Mikkelsen et al.^58^ recognized that glucose was found to be useful not only as a source of energy but also as an ideal precursor for the polymerization of cellulose by the bacteria *Gluconacetobacter xylinus*. On the other hand, Ishihara et al.^52^ investigated the use of D-xylose as a carbon source for biosynthesis of bacterial nanocellulose and concluded that xylose is poorly assimilated by any strain of bacteria that capable of producing substantial amounts of bacterial nanocellulose in glucose medium.

In the process of bacterial cellulose production, fruit juices were utilised as an alternative source of carbon^59^ such as those obtained from orange, water melon, pineapple, muskmelon, pomegranate, coconut milk, coconut water, tomato, apple and sugarcane juice. Extracts made from the skins and peels of many fruits, including watermelon, pineapple, banana, algarroba, grape, and Japanese pear^60^ were also used as culture medium for bacterial cellulose production. Culture medium for bacterial cellulose production was made using hydrolysates derived from several sources including sunflower zsmeal, wheat straw spruce, papers, and elephant grass; industrial by-products including beet molasses, sugar cane molasses^61^ soya bean whey, cheese whey and brewery waste^62^. In addition, agro-industrial wastes such as flour-rich wastes, coffee cherry husk, wine distillery waste, dry olive mill residues, and saccharified food wastes have been used for the production of bacterial cellulose as alternative carbon sources^59,63^. These raw materials typically contain a significant amount of a variety of sugars, including sucrose, fructose, lactose, glucose and xylose^63^.

### Identification of the selected strain no. SEE-3

**Figure 2:**
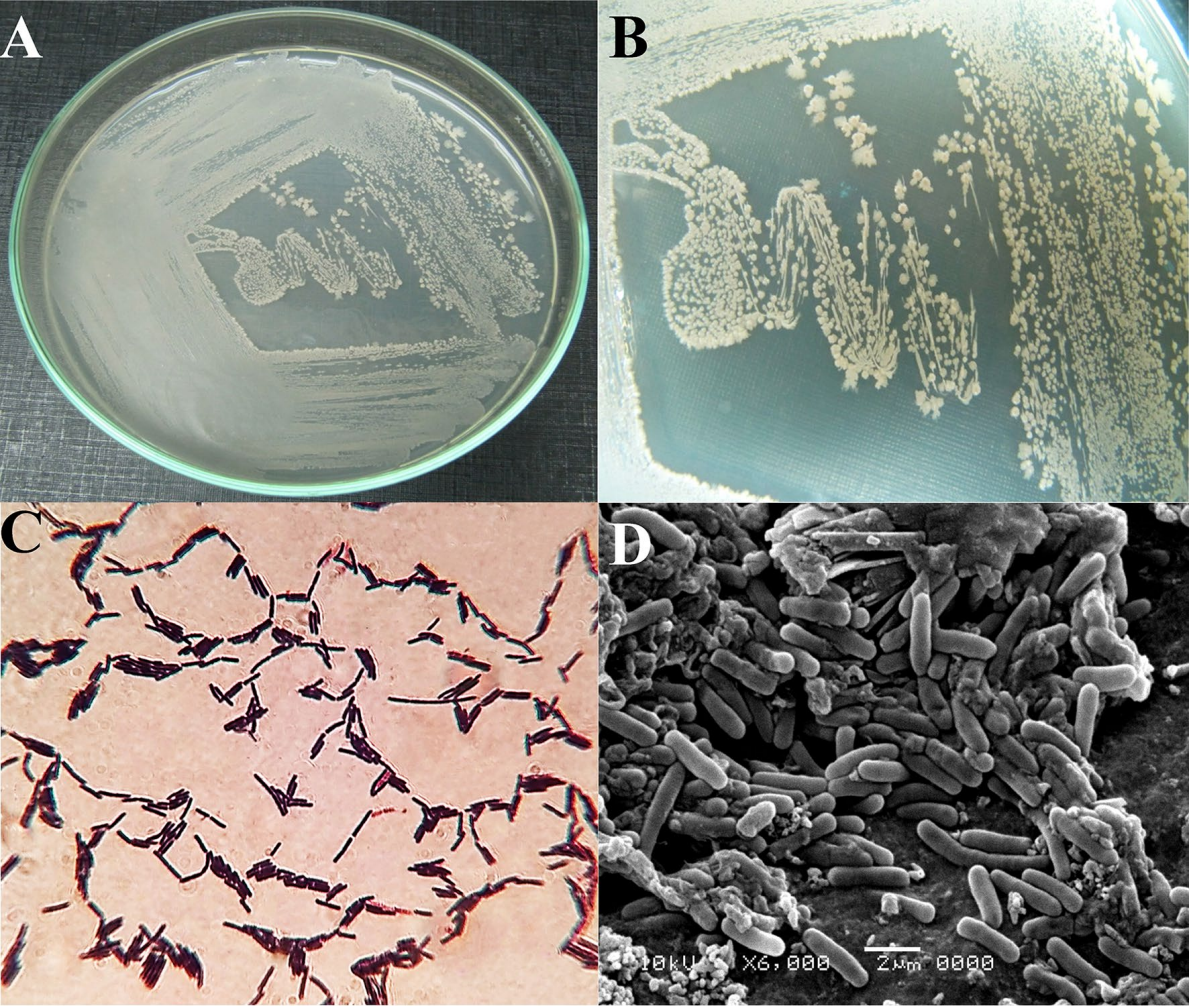
(**A**, **B**) Cultural characteristics of strain SEE-3 grown on nutrient agar plates. (**C**) Gram stain test shows Gram-positive bacilli. (**D**) Scanning electron micrograph showing cell morphology of strain SEE-3 at magnification of 6000 X.

The selected strain no. SEE-3 was characterized based on the taxonomic features, and 16S rRNA sequence analysis. Supplementary Table S2 show the main morphological and biochemical features of the strain no. SEE-3. They develop a flat growth pattern on the surface of the LB medium. Colonies of the strain no. SEE-3 (Fig. 2A,B) are large and irregular in shape, with undulated margin. The strain was found to be related to the *Bacillus* genus, according to the microscopic investigation. *Bacillus* sp*.* strain SEE-3 is aerobic, Gram‐positive (Fig. 2C), motile rods, produced oval spores. Scanning electron microscopy has revealed rod-shaped bacilli (Fig. 2D). The strain is characterized by their capacity to utilize glucose, fructose, mannitol, sucrose, galactose, sorbitol, raffinose, glycine and CMC. While ribose, lactose, mannose, trehalose and xylose were not utilized.

**Figure 3:**
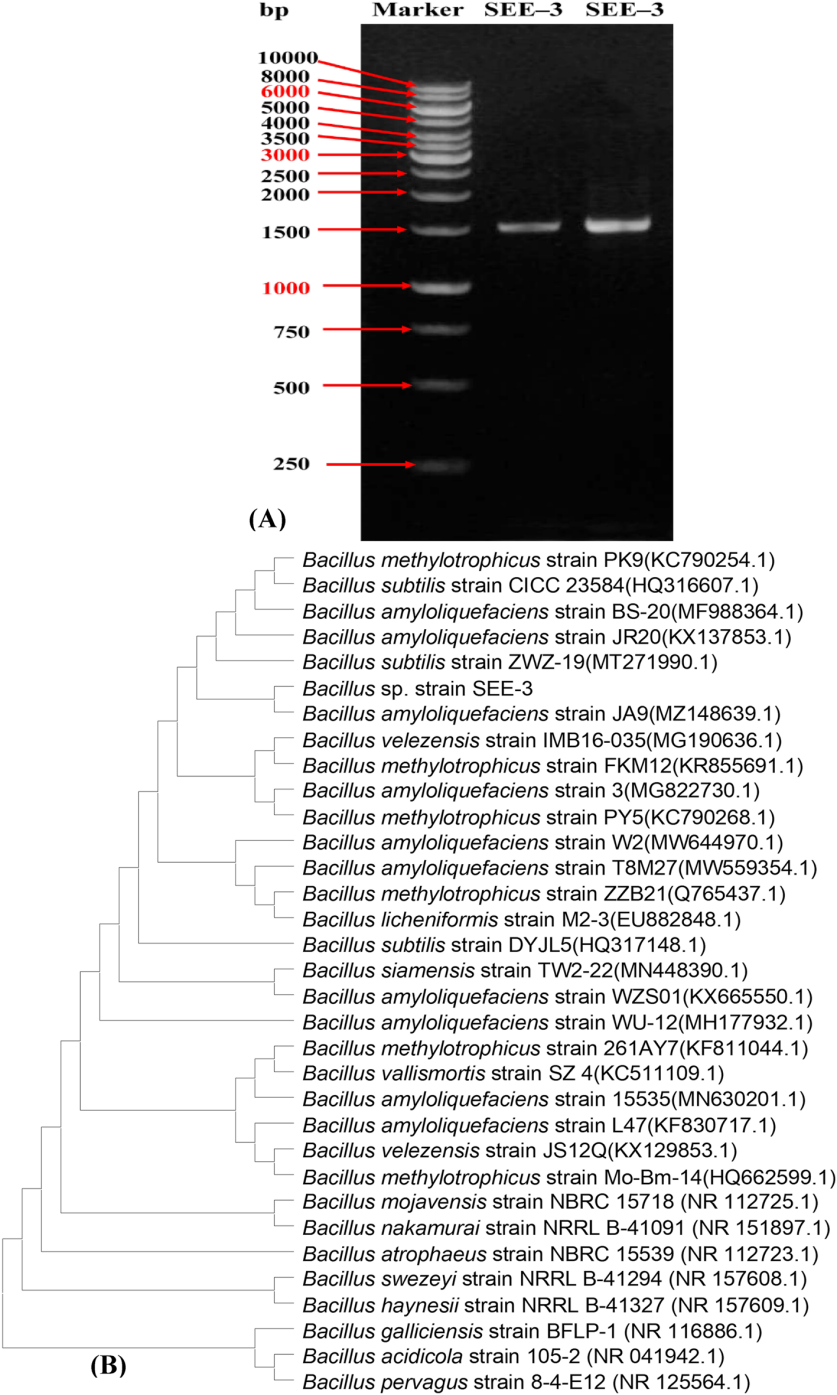
(**A**) Agarose gel electrophoresis showing the PCR product of the amplified 16S rRNA fragment for the strain SEE-3. (**B**) The phylogenetic tree of strain SEE-3 and related species of the genus *Bacillus*. (**B**) was created by using MEGA version X software.

The 16S rRNA gene sequence of *Bacillus* sp. strain SEE-3 was used to confirm the strain's identification. The obtained 16S rRNA fragment sequence was amplified by polymerase chain reaction (PCR) and the amplified segment displayed a distinct band of 1065 bp that corresponded to the sequencing product (Fig. 3A). For identification, the 16S rRNA gene sequence of *Bacillus* sp. strain SEE-3 was compared for similarity with the bacterial sequences deposited in GenBank using NCBI BLAST (available on http://www.ncbi.nlm.nih.gov/, accessed on 31 July 2022). The comparison gave similarity more than 98%, with the bacterial sequences of type strains.

A phylogenetic tree of *Bacillus* sp. strain SEE-3 (Fig. 3B) was conducted using the Maximum Parsimony method in MEGA version X software^45^. This tree shows the close phylogenetic association of *Bacillus* sp. strain SEE-3 with certain other *Bacillus* species. Phylogenetic analysis indicated that the strain SEE-3 consistently falls into a clade together with 98% sequence coverage in comparison for *Bacillus amyloliquefaciens* strain JA9(MZ148639.1) (98.48%), *Bacillus amyloliquefaciens* strain JR20 (accession No. KX137853.1, similarity 98.48%), *Bacillus amyloliquefaciens* strain BS-20 (accession No. MF988364.1, similarity 98.48%), *Bacillus methylotrophicus* strain PK9 (accession No. KC790254.1, 98.48%), *Bacillus subtilis* strain CICC 23584 (accession No. HQ316607.1, similarity 98.48%), *Bacillus subtilis* strain ZWZ-19 (accession No. MT271990.1, similarity 98.48%). Accordingly, strain SEE-3 was identified as *Bacillus* sp*.* strain SEE-3. The 16S rDNA gene sequence had been deposited to NCBI Gen Bank under the accession number of MN826326 (https://www.ncbi.nlm.nih.gov/nucleotide/MN826326.1?report=genbank&log$=nucltop&blast_rank=1&RID=MEKVRA6801R).

### Physical properties of bacterial nanocellulose

The purified powder of the bacterial nanocellulose was odorless, white to pale yellow color (Fig. 1C).

### Solubility in water and organic solvents

Solubility of the bacterial nanocellulose produced by *Bacillus* sp*.* strain SEE-3 was investigated in water, and standard organic solvents (acetic acid, xylene, DMSO, butanol, methanol, isopropanol, ethanol, propanol, chloroform), as well as ammonia solution and a mixture of 7% NaOH, 12% urea, and 81% distilled water. The bacterial nanocellulose produced by *Bacillus* sp*.* strain SEE-3 is insoluble in water, ammonia solution or organic solvents, which conferred with general characteristics of cellulose. Only, the mixture of 7% NaOH, 12% urea, and 81% distilled water can dissolve the bacterial nanocellulose produced by *Bacillus* sp*.* strain SEE-3. Because of its high polarity and strong intermolecular hydrogen bonding, bacterial nanocellulose is insoluble in water and other common organic solvents^46^.

### Scanning electron microscopy (SEM) and transmission electron microscopy (TEM) analyses

**Figure 4:**
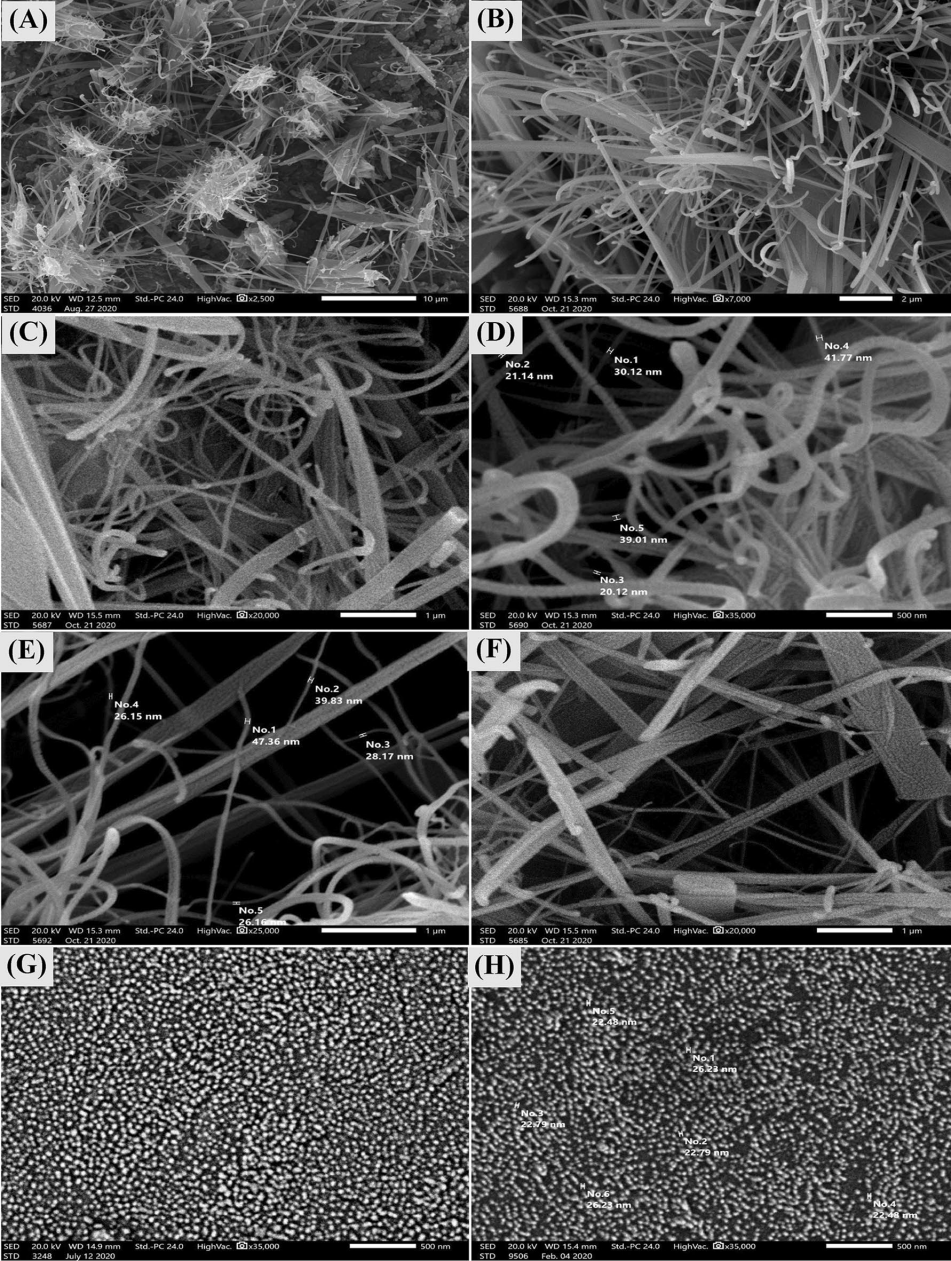
(**A**–**F**) Scanning electron microscopy morphology of bacterial nanocellulose sample soaked in 0.1 M NaOH for 1 h at room temperature to remove medium components and dissolve the bacteria cells possibly entrapped in the bacterial cellulose microfibers. (**G**, **H**) Scanning electron microscopy morphology of sonicated bacterial nanocellulose sample.

SEM analysis was carried out to characterize morphology and microstructure of the bacterial nanocellulose. SEM helps to determinate the structure and homogeneity of bacterial nanocellulose particles. Figure 4A–F show the SEM micrographs of the bacterial nanocellulose produced by *Bacillus* sp*.* strain SEE-3 on Cantaloupe juice after treatment process. The micrograph shows ultrafine threadlike microfibrils of cellulosic material that lack visible bacterial cells. Some microfibrils are separated while other fibrils are tightly packed . The SEM images shows fibers with diameters of 20.12‒47.36 nm and lengths of several nanometers. This confirms that the formation of fiber-shaped particles of bacterial nanocellulose using Cantaloupe juice.

**Figure 5:**
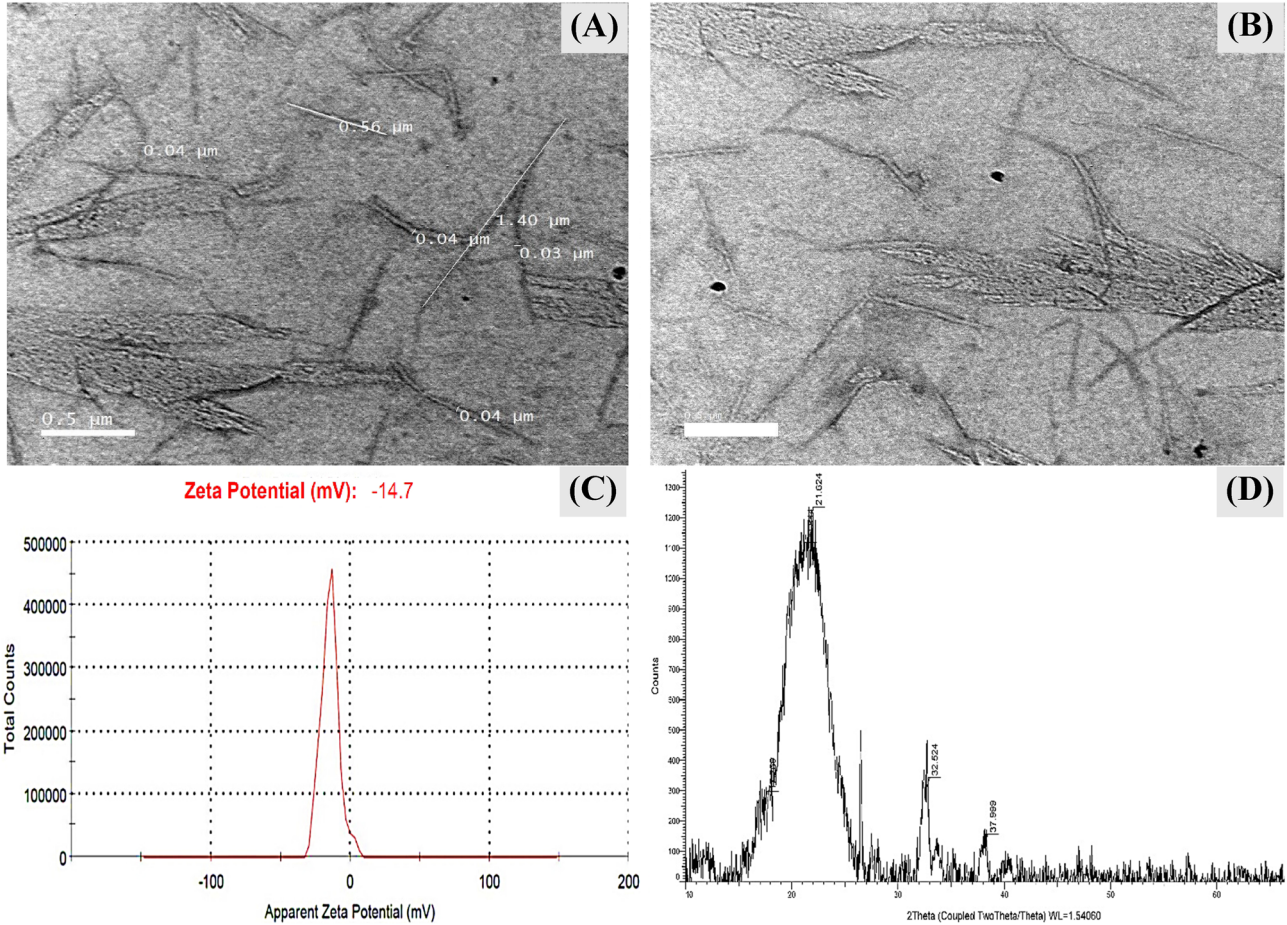
(**A**, **B**) Transmission electron microscopy morphology of bacterial nanocellulose sample, (**C**) Zeta potential, (**D**) X-ray diffraction of *Bacillus* sp*.* strain SEE-3.

Scanning electron microscopy (SEM) images of the sonicated bacterial nanocellulose sample produced by *Bacillus* sp*.* strain SEE-3 on Cantaloupe juice after treatment process (Fig. 4G,H) revealed crystalline clusters and spherical particles. Results demonstrated that, ultrasonic treatment produced small size particles and homogenous dispersion products. The SEM images (Fig. 4G,H) shows spherical nano-cellulose particles with diameters of 22.48‒26.23 nm. By sonication, the production of spherical nanocellulose can be achieved from nanocellulose fibers. Modification of cellulosic fibers using ultrasound has also been reported. Application of higher ultrasonication power caused the occurrence of a lot of small spherical particles^64^. The TEM images (Fig. 5A,B) shows needle-shaped particles with diameters of 30‒40 nm and lengths of 560‒1400 nm.

### Surface charge properties

The zeta potential (ζ) determines the surface charge properties of the bacterial cellulose nanofibers. As shown in Fig. 5C the cellulose nanofibers possess a negatively charged surface of − 14.7 mV. Generally, most cellulosic fibers tend to have a negative charge in water. Due to the presence of sulphate ester groups (OSO_3_^−^) on the surface of the bacterial cellulose nanocrystals, a negative zeta potential in the range of − 9.5 to − 15.5 mV was observed^65^. A zeta potential value of − 14.7 mV was significantly lower when compared to zeta potential values (ranging from − 33.1 to − 35.7 mV) of pristine cellulose nanocrystals derived from wood^66^. Lee et al.^67^ reported that a zeta potential value for fibrillated the bacterial cellulose was − 16.9 mV.

### X-ray diffraction (XRD)

In order to examine the crystallographic structure of the bacterial nanocellulose produced by *Bacillus* sp*.* strain SEE-3 from Cantaloupe juice fermentation, the XRD analysis was conducted. X-ray diffraction pattern of the bacterial nanocellulose sample has been shown in Fig. 5D. The XRD pattern revealed five visible peaks shown in the entire diffractogram located at 2*θ* values of 17.26°, 20.84°, 21.62°, 32.52° and 37.99°. XRD diffractograms of the microcrystalline cellulose, typical diffractions due to cellulose I are observable at 2*θ* = 17° and 32°, which correspond to ($10\overline{1}$) and (040); respectively^68^ Peaks located at 2*θ* values of 20, and 37 correspond to crystallographic planes cellulose II^69^. However, Gong et al.^70^ reported that the characteristic peak for the cellulose type-III was recorded at 2*θ* = 21° that comprises the planes (100), (012) and (1–10).

Results show that the bacterial nanocellulose obtained from Cantaloupe juice fermentation has crystallinity degree value of 79.58%. It is possible to determine the crystallinity of cellulose using a variety of methods, and it is well established that the results are dependent on the technique used^71^. Crystallinity is a significant characteristic of nanocellulose, which defines its physical and mechanical characteristics and this has a powerful impact on the final application of the nanoparticles^72^ There are four different crystalline polymorphic (the unit has different dimensions) structures in cellulose (types I, II, III and IV). Cellulose type I is a fundamental crystalline structure that found in a broad range of cellulosic fibers. Cellulose type I can be used for hydrogel synthesis with improved mechanical properties^73^.

NaOH treated cellulose displayed peaks typical of cellulose II polymorph at 2θ = 20°, 22°, and 37° correspond to the ($10\overline{1}$), (002), and (040) crystallographic planes, respectively. Whereas, no peaks are found at 2*θ* = 20.8°, which are characteristic of cellulose II^74^. The diffractograms for microcrystalline cellulose display diffraction patterns that are typical of cellulose. The diffraction peaks of the 2*θ* angles are located at 20.2 and 21.90 degrees^75^. The peak of cellulose II can be located at 2*θ* = 20.5° and is assigned to the (1–10) plane^76^.

Cellulose I is a type of crystalline cellulose that is formed in nature by a wide variety of organisms (such as bacteria, algae, tunicates, plants and trees), and it is composed of parallel chains^77^. The cellulose I structure is thermodynamically metastable and can be transformed into cellulose II or III. All cellulose strands are arranged in a highly ordered parallel configuration^74^. Cellulose I can be transformed to cellulose II through two distinct processes: regeneration (also known as solubilization and recrystallization) and mercerization by alkaline solution. Cellulose I and Cellulose II can be converted into Cellulose III using thermal treatments and liquid ammonia. Cellulose IV is produced with certain treatments of Cellulose III^78^.

### FTIR spectra of the bacterial nanocellulose and Avicel PH101

**Figure 6:**
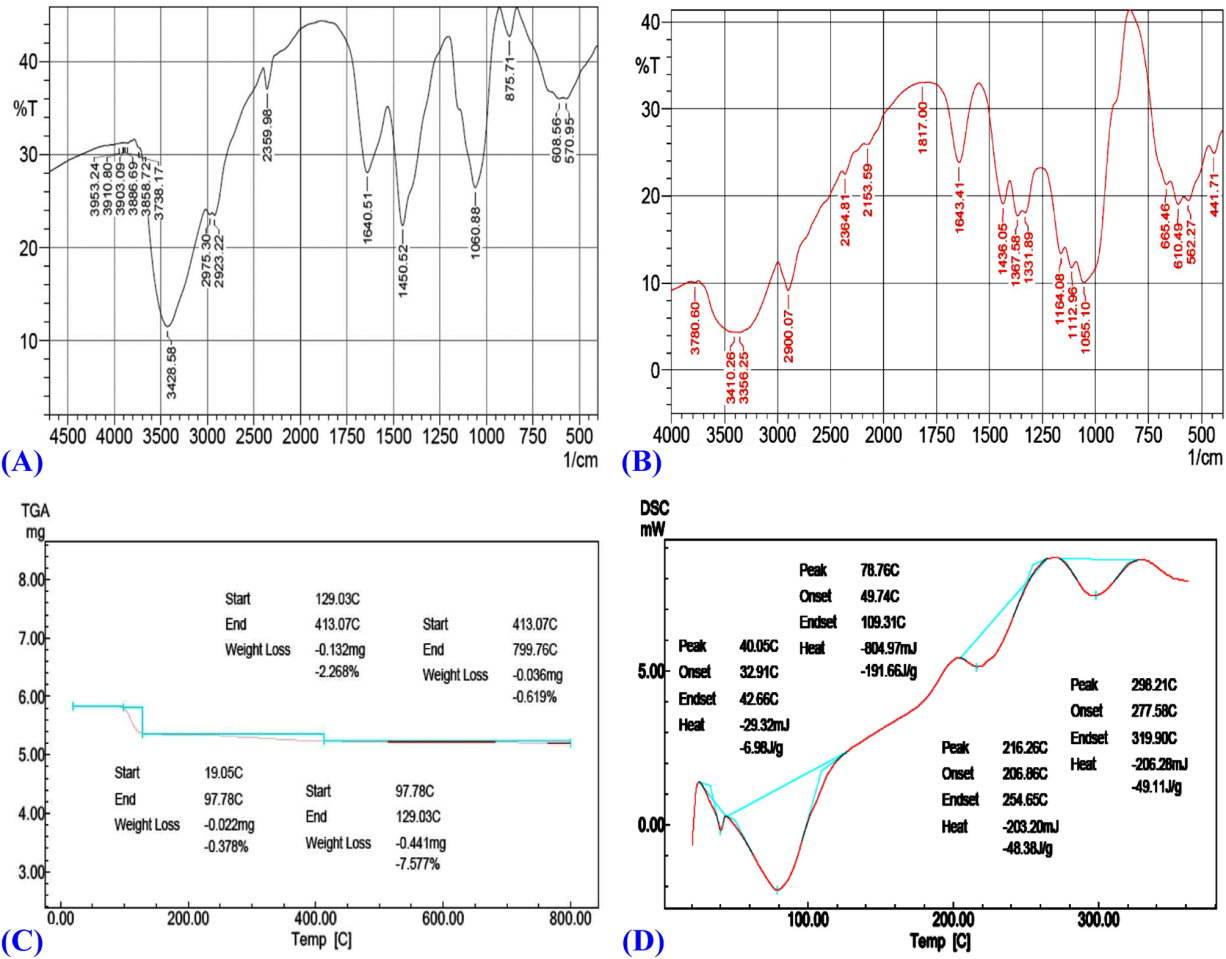
FTIR spectra of: (**A**) *Bacillus* sp*.* strain SEE-3 bacterial nanocellulose; and (**B**) Avicel PH101, (**C**) Thermogravimetric analysis (TGA), and (**D**) DSC analysis for *Bacillus* sp*.* strain SEE-3 bacterial nanocellulose.

FTIR spectroscopy is a powerful tool for studying the physicochemical properties of polysaccharides. Figure 6A,B shows the FTIR spectra in 4000–500 cm^−1^ region of the bacterial nanocellulose and standard cellulose (Avicel PH-101) from Sigma-Aldrich for analysis of functional groups present in their structure. Both of the FTIR curves have the typical structure of cellulose, with the exception of a few differences in the bacterial nanocellulose spectrum compared to the standard cellulose spectrum. Bands in the 400–700 cm^−1^ range characteristics of the O–H bending^79^. Gupta et al.^80^ and Fu et al.^25^ reported that the peaks at 893‒1105 cm^−1^ corresponds to the stretching vibration of β-glycosidic linkage at cellulose ring. In addition, β-glycosidic linkage of cellulose ring for γ (COC) in-plane, symmetric stretching assigned at 869.92 cm^−1^^81^. The peaks in the range of lower than 900 cm^−1^ is attributed to C–OH and C–C bending^82^.

The spectra revealed the characteristic bands of the cellulose crystal structure by the presence of peak for the bacterial nanocellulose at 1060 cm^−1^ assigned to C–O–C group of carbohydrate skeleton, which shifted to 1055 cm^−1^ for Avicel PH101. Besides that, the peaks at 1060 cm^−1^ in pure bacterial nanocellulose can be attributed to vibration of β-glycosidic linkage at bacterial nanocellulose ring (C–O)^25,82^. The bands at 1112 and 1164 cm^−1^ assigned to C–C bonds in polysaccharide monomer units or C–O bending vibration, 1100–1350 corresponds to acyl pheny and C–O, C–O–C antisymmetric bridge stretching of the ether linkage of cellulose (1, 4-β-d-glucoside)^83,84^. The band at 1640 cm^−1^ can be attributed to the N–H group derived from the amide I of bacterial cellulose protein. The bacterial cellulose and Avicel PH101 samples have the peaks around 2900 cm^−1^ attributed to the C–H stretching, because of the presence of the CH and CH_2_ groups in both the cellulose and Avicel PH101.

The peak around 2900 cm^−1^ represents amorphous nature. Also, the peak at 2975 cm^−1^ is assigned to the C‒H/CH_2_ stretching vibrations^85^. As well as, in pure bacterial nanocellulose, a broad band at 3428 cm^−1^ is assigned to O–H stretching vibration, which shifted to 3410 cm^−1^ in Avicel PH101. The region between 3200 and 3550 cm^−1^ reflects the stretching vibration of O–H bond (hydroxyl groups)^80,86^. Because cellulose is a polysaccharide, it contains a large number of OH groups. The O–H stretching vibration of pure cellulose is responsible for the extremely strong absorption band that occurs at 3410 cm^−1^^87^. The peaks around in 3400–3500 cm^−1^ is attributed to O–H stretching for pure cellulose^87^. The bands at 3858, 3886, 3903, 3910 and 3953 cm^−1^ corresponds to O–H stretching^88^. 

Table 1 shows the features of bacterial cellulose produced by strain SEE-3 in comparison with other bacterial species^39,89–95^.

### Thermogravimetric analysis (TGA)

The thermogravimetric degradation curve of the dried bacterial nanocellulose in percentage of the mass in the initial sample depending on the temperature. The sample showed slight weight loss during the initial thermal treatment (Fig. 6C) from room temperature (19.05 °C) to 97.78 °C. A second, substantial weight loss occurred with 97.78–129.03 °C, which may be attributable to the loss of water. While the bacterial nanocellulose showed a very low mass loss rate of 0.619% at 413.07–799.76 °C, which could be caused by cellulose degradation including the dehydration, de-polymerization and glucose units decomposition. These results conferred high thermal stability to the bacterial nanocellulose produced by *Bacillus* sp*.* strain SEE-3 using Cantaloupe juice. Thermal stability of the bacterial cellulose produced by the strain SEE-3 has been compared with other bacterial nanocellulose (Table 1)^89–95^. The bacterial nanocellulose produced by SEE-3 had greater thermal stability, as a very low mass loss rate of 0.619% began at 413.07–799.76 °C, while the BC (nata de coco) produced by *Komagataeibacter xylinus* showed a mass loss rate of 0.77% at 335°C^89^. The BC produced by *Komagataeibacter rhaeticus* PG2 showed a weight loss of 85–95% up to 395°^90^. On the other hand, the BC produced by *Acetobacter xylinum* showed a quick drop in sample weight begins at 300 °C, the maximum decomposition occurs at 350–370 °C^91^. The BC produced by *Komagataeibacter hansenii* showed 70–80% of weight loss at 360–600 °C^95^. Thermal stability is assessed by the maximum decomposition temperature. Thermal degradation is influenced by structural factors such as crystallinity, the way in which the fibres are arranged and molecular mass^74^.

**Table 1:** Features of the bacterial cellulose produced by the strain SEE-3 in comparison with other bacterial species.

BC source	Properties	Structure and properties	References
*Bacillus* sp*.* strain SEE-3	Morphology	BC is needle-shaped particles with diameters of 30‒40 nm and lengths of 560‒1400 nm	Current study
	Crystallinity	Crystallinity degree value of 79.58%	
	Zeta potential	Possess a negatively charged surface of Zeta potential − 14.7 mV	
	Solubility	Insoluble in water, ammonia solution or organic solvents. Soluble in the mixture of 7% NaOH, 12% urea, and 81% distilled water	
	Maximum weight loss temperature	The bacterial nanocellulose showed a very low mass loss rate of 0.619% at 413.07–799.76 °C (TGA analysis)	
BC (nata de coco) *Komagataeibacter xylinus*	Morphology	BC is needle-like with diameters ranging between 33.7 and 44.3 nm, and lengths ranging between 622 and 1322 nm	Vasconcelos et al.^89^
	Crystallinity	Crystallinity degree value of 79%	
	Zeta potential	Possess a negatively charged surface	
	Solubility	Not detected	
	Maximum weight loss temperature	The bacterial nanocellulose showed a mass loss rate of 0.77% at 335 °C (TGA analysis)	
*Komagataeibacter rhaeticus* PG2	Morphology	BC is a fibrillar width in the range of 30–80 nm	Thorat and Dastager^90^
	Crystallinity	Crystallinity degree value of 58–80%	
	Zeta potential	Not detected	
	Solubility	Not detected	
	Maximum weight loss temperature	The maximum degradation of weight loss is 85–95% was observed up to 395 °C (TGA analysis)	
*Acetobacter xylinum*	Morphology	Long, smooth and oriented fibril bundles s that have a width varying from 70 to 200 nm	Surma-Ślusarska et al.^91^
	Crystallinity	Not detected	
	Zeta potential	Not detected	
	Solubility	Not detected	
	Maximum weight loss temperature	A quick drop in sample weight begins at 300 °C, the maximum decomposition occurs at 350–370 °C	
*Gluconacetobacter hansenii* (*Komagataeibacter hansenii*)	Morphology	Ultrafine nanofibrils, average fiber diameters were ranged from 47.64 to 61.11 nm	Costa et al.^39^, Güzel and Akpınar^95^
	Crystallinity	crystallinities were ranged from 80.27 to 92.96%	
	Zeta potential	Not detected	
	Solubility	Not detected	
	Maximum weight loss temperature	Maximum decomposition occurred at 290–310 °C based on the medium. 70–80% of weight loss is observed at 360–600 °C	
*Gluconacetobacter xylinus*	Morphology	A dense network of fibrils with an average diameter of 30–200 nm	Sijabat et al.; Abba et al.; Nyakuma et al.^92–94^
	Crystallinity	The crystallinity index was 86.94%	
	Zeta potential	The potential zeta absolute value − 11.39 mV	
	Solubility	Not detected	
	Maximum weight loss temperature	Significant thermal degradation from 183.24 to 363.79 °C and total mass loss was 57.24	

### Differential scanning calorimetry (DSC) analysis

Figure 6D shows the DSC curve obtained from the bacterial nanocellulose produced by *Bacillus* sp*.* strain SEE-3. It can be seen from the DSC curve that the bacterial nanocellulose sample contained four peaks. The first transformation peak at 40.05 °C is the thermal effect of dehydration and water loss from the sample. The second peak at 78.76 °C can be attributed to dehydration and water loss or the melting of cellulose's crystalline phase. Auta et al.^96^ reported that the initial bacterial cellulose sample varied greatly between 10 and 200 °C due to water content evaporation. Whereas, George et al.^97^ reported that there is a known transformation related to the melting of the crystalline phase of cellulose at temperatures ranging from 80 to 140 °C. An endothermic peak around 40–100 °C is observed due to dehydration and water loss^98^. The third peak at 216.26 °C The fourth peak at 298.21 °C can be attributed to glass transition (T*g*) and crystallization. The glass transition observed at 270 °C, and crystallisation observed at 330°C^98^. The glass transition is the gradual and reversible transformation in amorphous solid region from a rigid and fairly glassy state into a rubbery and less viscous state with the rise in temperature. Mishra et al.^99^ reported that the viscosity of an amorphous solid polymer decreased as the temperature increased, and at a certain temperature (crystallisation temperature), the particles became more mobile and organised into a crystalline solid polymer through an exothermic process. The crystallinity and high molecular weight of bacterial cellulose contribute to its thermal stability up to 200 °C^100^. Whereas, the low thermal stability of bacterial cellulose may be due to hydrolysis producing low molecular weight oligosaccharides.

### Plackett–Burman design to identify significant factors affecting bacterial nanocellulose production by *Bacillus *sp*.* strain SEE-3

In the present study, the influence of ten nutritional and environmental factors were evaluated for their effects on the bacterial nanocellulose production using a Plackett–Burman experimental design including: A (medium volume; mL/250 mL conical flask), B (pH), C (incubation time; days), D (inoculum size; %, v/v), E (Cantaloupe juice; %, v/v), F (citric acid; g/L); G (peptone; g/L), H (yeast extract; g/L), J (temperature; °C), K (Na_2_HPO_4_; g/L) in addition to one dummy variable. Table 2 illustrates the 12-run Plackett–Burman experimental design matrix that was used to screen for significant factors influencing bacterial nanocellulose synthesis and the resultant bacterial nanocellulose production. The bacterial nanocellulose production varied markedly from 0.39 to 14.81 g/L (Table 2). This variance showed the significance of process optimization in achieving maximum bacterial nanocellulose production. The results showed that the lowest value of the bacterial nanocellulose production (0.39 g/L) was achieved in run no. 2 when the independent factors were: A (medum volume; 50 mL/250 mL conical flask), B (pH 3.6), C (incubation time; 14 days), D (inoculum size; 5%, v/v), E (Cantaloupe juice; 100%, v/v), F (citric acid; 2.67 g/L), G (peptone; 5 g/L), H (yeast extract; 10 g/L), J (temperature; 37 °C), K (Na_2_HPO_4_; 5 g/L). While, the highest value of the bacterial nanocellulose production (14.81 g/L) was achieved in the run no. 12 when the independent factors were: A (medium volume; 100 mL/250 mL conical flask), B (pH 5), C (incubation time; 14 days), D (inoculum size; 5%, v/v), E (Cantaloupe juice; 75%, v/v), F (citric acid; 1.5 g/L), G (peptone; 10 g/L), H (yeast extract; 5 g/L), J (temperature; 37 °C), K (Na_2_HPO_4_; 5 g/L) were used.

**Table 2:** Plackett–Burman experimental design for evaluation of independent factors for the production of bacterial nanocellulose by *Bacillus* sp*.* strain SEE-3 with coded values along with the experimental bacterial nanocellulose.

Std no	Run no	A	B	C	D	E	F	G	H	J	K	Dry wt. of bacterial nanocellulose (g/L)		Residuals
												Actual	Predicted	
1	1	1	1	− 1	1	1	1	− 1	− 1	− 1	1	4.09	4.24	− 0.15
5	2	− 1	− 1	1	− 1	1	1	− 1	1	1	1	0.39	0.535	− 0.15
4	3	− 1	1	− 1	1	1	− 1	1	1	1	− 1	6.33	5.99	0.34
11	4	1	− 1	1	1	1	− 1	− 1	− 1	1	− 1	3.92	3.78	0.15
6	5	− 1	− 1	− 1	1	− 1	1	1	− 1	1	1	9.44	9.78	− 0.34
8	6	1	1	− 1	− 1	− 1	1	− 1	1	1	− 1	10.72	10.58	0.15
3	7	1	− 1	1	1	− 1	1	1	1	− 1	− 1	9.4	9.06	0.34
10	8	− 1	1	1	1	− 1	− 1	− 1	1	− 1	1	5.3	5.64	− 0.34
7	9	1	− 1	− 1	− 1	1	− 1	1	1	− 1	1	1.1	1.44	− 0.34
12	10	− 1	− 1	− 1	− 1	− 1	− 1	− 1	− 1	− 1	− 1	2.15	1.81	0.34
2	11	− 1	1	1	− 1	1	1	1	− 1	− 1	− 1	5.52	5.38	0.15
9	12	1	1	1	− 1	− 1	− 1	1	− 1	1	1	14.81	14.96	− 0.15

Variable level	*		Days	%, v/v	%, v/v	g/L	g/L	g/L	°C	g/L				
− 1	50	3.6	7	5	75	1.5	5	5	30	3				
1	100	5	14	10	100	2.67	10	10	37	5				

The independent factors are: A * (medium volume; mL/250 mL conical flask), B (pH), C (incubation time; days), D (inoculum size; %, v/v), E (Cantaloupe juice; %, v/v), F (citric acid; g/L); G (peptone; g/L), H (yeast extract; g/L), J (temperature; °C), K (Na_2_HPO_4_; g/L).

### Statistical evaluation of the Plackett–Burman design for *Bacillus *sp*.* strain SEE-3 bacterial nanocellulose production

**Figure 7:**
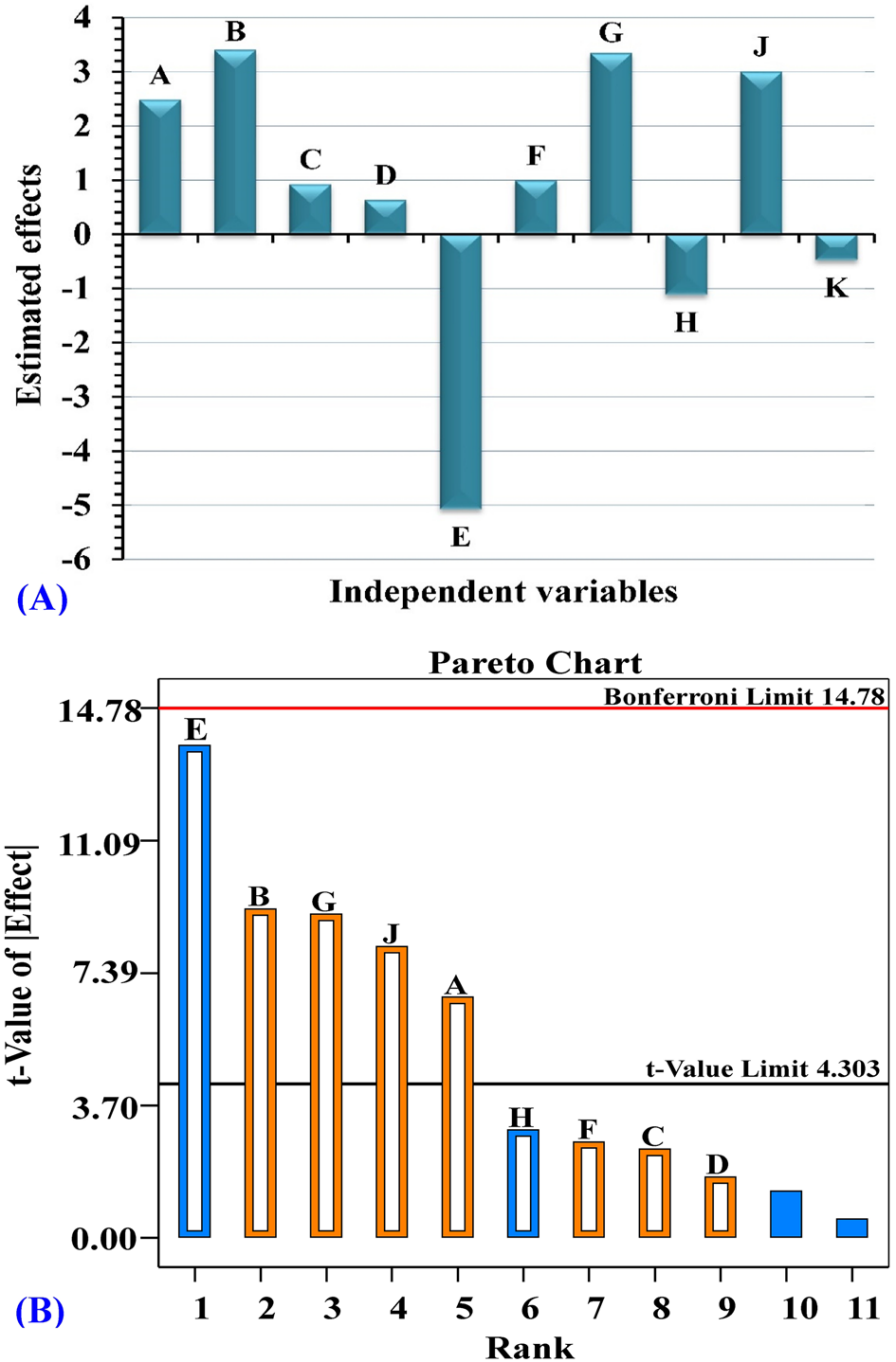
(**A**) Estimated effects of independent factors, The independent factors are: A (medium volume; mL/250 mL conical flask), B (pH), C (incubation time; days), D (inoculum size; %, v/v), E (Cantaloupe juice; %, v/v), F (citric acid; g/L); G (peptone; g/L), H (yeast extract; g/L), J (temperature; °C), K (Na_2_HPO_4_; g/L). (**B**) Pareto chart shows the order and significance of the factors on the bacterial nanocellulose production by *Bacillus* sp*.* strain SEE-3 using Plackett–Burman design “the blue colors represent negative effects and the orange color represent positive effects”. This figure was created by using Design Expert version 12 for Windows software.

Multiple-regression statistical analysis of the Plackett–Burman design results and analysis of variance (ANOVA) were calculated and shown in Table 3 to assess the correlation between the bacterial nanocellulose production by *Bacillus* sp*.* strain SEE-3 and the independent variables. Table 3 and Fig. 7A show the coefficients estimate and effect of each independent variable on the bacterial nanocellulose production. The signs of the coefficients and effects were used to interpret the data. The factor has a significant influence on the response if the effect is large, regardless of whether it is positive or negative^101,102^. When the effect of a tested factor is positive, it means that the production is increased at a high level of the factor. On the other hand, when the sign is negative, it means that the production is higher when the factor level is low. According to the regression coefficients and the estimated effects, seven of the ten parameters (medium volume, pH, incubation time, inoculum size, citric acid, peptone, temperature) have a positive effect on nanocellulose production (Table 3, Fig. 7A); the other three factors (Cantaloupe juice, yeast extract, Na_2_HPO_4_) have a negative effect on the production of nanocellulose. Table 3 also shows the percentages of each variable's contribution. Among the independent factors, pH (B), Cantaloupe juice (E) and peptone (G) with estimated effects values of 3.4, − 5.08 and 3.34 and percent of contribution 17.09, 38.25 and 16.53%; respectively, suggesting that these factors could have a large impact on the production of bacterial nanocellulose.

**Table 3:** Statistical analysis of Plackett–Burman design showing coefficient estimate, effect, % contribution, *F*-value and *P* value for each factor affecting bacterial nanocellulose production by *Bacillus* sp*.* strain SEE-3.

Term	*df*	Coefficient estimate	Effect	% Contribution	*F*-value	*P* value
Model	1	6.10			54.61	0.0181*
A-Medium volume	1	1.24	2.48	9.16	45.20	0.0214*
B-pH	1	1.70	3.40	17.09	84.36	0.0116*
C-Incubation time	1	0.46	0.92	1.25	6.17	0.1309
D-Inoculum size	1	0.32	0.63	0.59	2.92	0.2296
E-Cantaloupe juice	1	− 2.54	− 5.08	38.25	188.76	0.0053*
F-Citric acid	1	0.50	0.99	1.46	7.20	0.1154
G-Peptone	1	1.67	3.34	16.53	81.57	0.012*
H-Yeast extract	1	− 0.56	− 1.12	1.84	9.10	0.0946
J-Temperature	1	1.50	3.00	13.42	66.24	0.0148*
K-Na_2_HPO_4_	1	− 0.24	− 0.48	0.35	54.61	0.24

Std. Dev.	0.64	R-Squared	0.9959			
Mean	6.10	Adj R-Squared	0.9777			
C.V.%	10.50	Pred R-Squared	0.8541			
PRESS	29.51	Adeq Precision	24.67			

*Significant values, *F*: Fishers's function, *P*: Level of significance, C.V: Coefficient of variation.

The determination coefficient (R^2^) value indicates the extent to which the independent variables can explain the observed response values. R^2^ always has a value between 0 and 1. The design used is more precise for predicting the response when the determination coefficient (R^2^) value is closer to 1^103^. The R^2^ value in this study is 0.9959, indicating that the model is fit and able to provide an explanation of 99.59% of the variability in the bacterial nanocellulose production by *Bacillus* sp*.* strain SEE-3. The adjusted R^2^ of 0.9777 is extremely high and clarified the significance of the model (Table 3). Furthermore, the predicted-R^2^ value of 0.8541 is high and coincides reasonably well with the adjusted-R^2^ value of 0.9777, confirming the model's statistical validity and accurcy for the bacterial nanocellulose production by *Bacillus* sp*.* strain SEE-3. The Adjusted-R^2^ and predicted-R^2^ values must be within 20% of each other, so that we can say that there an adequate agreement between them and indicating that the model is of high significance and accuracy^104^.

To determine the model's and variables' significance, *P* and *F*-values were calculated (Table 3). Variables that have confidence levels greater than or equal to 95% (*P* values less than or equal to 0.05) are regarded as significant^105^. The most significat factors were Cantaloupe juice (*F*-value of 188.76 and *P* value = 0.0053), followed by pH (*P* value = 0.0116 and *F*-value of 17.09) and peptone (*F*-value of 81.57 and *P* value = 0.012). The model's *F*-value of 54.61 and a very low probability value (*P* value = 0.0181) (Table 3) indicates that it is highly significant. The data revealed that incubation time, inoculum size, citric acid, yeast extract and Na_2_HPO_4_ are non-significant independent factors (*P* ˃0.05) with lower effects (0.92, 0.63, 0.99, − 1.12 and − 0.48; respectively) and lower percent of contribution (1.25, 0.59, 1.46, 1.84 and 0.35; respectively). The model showed PRESS, mean, standard deviation and coefficient of variation percent values of 29.51, 6.10, 0.64 and 10.50; respectively (Table 3). The signal-to-noise ratio is determined by the adequate precision value; it is desirable to have a value that is greater than 4 since this indicates a strong model fit^106^. The current model's adequate precision value is 24.67, indicating that it can be used for design space navigation.

The relationship between the *t*-value (the absolute values of the standardized effects) and the ranks is shown in a Pareto chart (Fig. 7B). The Pareto chart reveals significance and magnitude of the factors that influence the nanocellulose production, depends on the significance level (Fig. 7B). On the Pareto chart, the effects that are greater than the *t*-value limit are significat.

The regression coefficients data was fitted to the first-order polynomial equation to describe the relationship between the independent factors and the bacterial nanocellulose production by *Bacillus* sp*.* strain SEE-3 in terms of the coded independent factors:

4 $$\begin{aligned} {\mathbf{Y}} & = + 6.1 + 1.24{\text{A}} + 1.70{\text{B}} + 0.46{\text{C}} + 0.32{\text{D}} - 2.54{\text{E}} + 0.5{\text{F}} \\ & \quad + 1.67{\text{G}} - 0.56{\text{H}} + 1.50{\text{J}} - 0.24{\text{K}} \\ \end{aligned}$$

where Y is the bacterial nanocellulose production by *Bacillus* sp*.* strain SEE-3, and the independent factors are: A (medium volume; mL/250 mL conical flask), B (pH), C (incubation time; days), D (inoculum size; %, v/v), E (Cantaloupe juice; %, v/v), F (citric acid; g/L); G (peptone; g/L), H (yeast extract; g/L), J (temperature; °C), K (Na_2_HPO_4_; g/L).

The parameters estimated to be optimal for maximum production of the bacterial nanocellulose by *Bacillus* sp*.* strain SEE-3 were used in a confirmation experiment to determine the precision of Plackett–Burman design. They were as follows: A (medium volume; 100 mL/250 mL conical flask), B (pH 5), C (incubation time; 14 days), D (inoculum size; 5%, v/v), E (peptone; 10 g/L), F (citric acid; 1.5 g/L); G (Cantaloupe juice; 75%, v/v), H (yeast extract; 5 g/L), J (temperature; 37 °C), K (Na_2_HPO_4_; 3 g/L). Under these conditions, the maximum production of the bacterial nanocellulose was 14.81 g/L which is higher than the bacterial nanocellulose gained prior to the use of Plackett Burman (9.4 g/L) by 1.58 times.

### Optimization of the bacterial nanocellulose production by *Bacillus *sp*.* strain SEE-3 using FCCCD

On the basis of the effects and *P* values (Table 4), the appropriate levels of the most significant independent factors including pH (X_1_), peptone concentration (X_2_), and Cantaloupe juice concentration (X_3_) as well as their mutual impacts on the nanocellulose production by *Bacillus* sp*.* strain SEE-3 were determined by further optimization using FCCCD. Other variables were set at their optimal points of Plackett–Burman design. Table 4 shows the FCCCD design matrix for 20 experimental runs used to optimize these variables and their concentrations at various coded and actual levels. The central point was replicated six times (2, 8, 10, 16, 17 and 18). Table 4 shows also nanocellulose production (predicted and experimental values), as well as the residual values. The amount of bacterial nanocellulose produced varies significantly depending on the levels of the fermentation process factors in both the experimental and predicted results. The results demonstrated significant diversity in the nanocellulose production by *Bacillus* sp*.* strain SEE-3 based on the levels of the three independent variables (Table 4). The central run no. 16 had the maximum bacterial nanocellulose production, with a value of 19.97 g/L at pH 5, peptone concentration of 10 g/L and 75% Cantaloupe juice concentration. While in the run no. 4, where pH was 4.5, peptone concentration of 5 g/L and Cantaloupe juice was 50%, the minimal bacterial nanocellulose production (4.52 g/L) was obtained.

**Table 4:** Face centered central composite design for evaluation of independent factors with coded values along with the experimental bacterial nanocellulose by *Bacillus* sp*.* strain SEE-3.

Std	Run	Type	Variables			Dry weight of bacterial cellulose (g/L of medium)		Residuals
			X_1_	X_2_	X_3_	Actual value	Predicted value	
4	1	Fact	1	1	− 1	8.96	8.81	0.15
16	2	Center	0	0	0	19.45	19.01	0.44
5	3	Fact	− 1	− 1	1	9.31	9.33	− 0.01
1	4	Fact	− 1	− 1	− 1	4.52	4.56	− 0.04
14	5	Axial	0	0	1	16.05	16.62	− 0.57
6	6	Fact	1	− 1	1	10.87	10.74	0.14
8	7	Fact	1	1	1	12.44	12.27	0.17
19	8	Center	0	0	0	18.94	19.01	− 0.07
9	9	Axial	− 1	0	0	16.50	16.72	− 0.21
17	10	Center	0	0	0	18.47	19.01	− 0.54
3	11	Fact	− 1	1	− 1	7.92	7.92	0.00
10	12	Axial	1	0	0	17.55	17.87	− 0.32
7	13	Fact	− 1	1	1	15.98	15.71	0.27
13	14	Axial	0	0	− 1	12.54	12.51	0.03
2	15	Fact	1	− 1	− 1	10.16	10.30	− 0.14
18	16	Center	0	0	0	19.97	19.01	0.96
20	17	Center	0	0	0	19.40	19.01	0.39
15	18	Center	0	0	0	18.90	19.01	− 0.11
11	19	Axial	0	− 1	0	14.95	14.90	0.05
12	20	Axial	0	1	0	16.76	17.35	− 0.58

Variable	Variable code	Coded and actual levels						
		− 1	0	1				
pH	X_1_	4.5	5	5.5				
Peptone (g/L)	X_2_	5	10	15				
Cantaloupe juice (%, v/v)	X_3_	50	75	100				

### Multiple regression analysis and ANOVA

Table 5 and Supplementary Table S3 show the results of the multiple regression analysis of the FCCCD experimental data as well as the results of the analysis of variance (ANOVA). It is thought that a regression model with an R^2^-value that is greater than 0.9 proves a very high degree of correlation^107^. R^2^-value of 0.9936 indicates that the model is capable of explaining 99.36% of the variation in bacterial nanocellulose production. The adjusted R^2^ value was found to be 0.9878, which is very high, implying that the predicted and experimental values of the bacterial nanocellulose production are quite similar. The predicted R^2^ value of 0.9774 is also high indicating the model's adequacy for predictions of the bacterial nanocellulose production by *Bacillus* sp*.* strain SEE-3 (Table 5).

**Table 5:** Statistical analysis for FCCCD of bacterial nanocellulose production by *Bacillus* sp*.* strain SEE-3.

Source of variance		Sum of squares	*df*	Mean square	*F-*value	*P* value *p*rob > *F*	Coefficient estimate
Model		394.45	9	43.83	171.31	< 0.0001*	19.01
Linear effects	X_1_	3.31	1	3.31	12.92	0.0049*	0.58
	X_2_	15.02	1	15.02	58.69	< 0.0001*	1.23
	X_3_	42.27	1	42.27	165.23	< 0.0001*	2.06
Interaction effects	X_1_ X_2_	11.77	1	11.77	46.01	< 0.0001*	− 1.21
	X_1_ X_3_	9.38	1	9.38	36.68	0.0001*	− 1.08
	X_2_ X_3_	4.57	1	4.57	17.87	0.0018*	0.76
Quadratic effects	X_1_^2^	8.12	1	8.12	31.72	0.0002*	− 1.72
	X_2_^2^	22.94	1	22.94	89.65	< 0.0001*	− 2.89
	X_3_^2^	54.43	1	54.43	212.75	< 0.0001*	− 4.45
Error effects	Lack of fit	1.16	5	0.23	0.83	0.5775	
	Pure error	1.40	5	0.28			

R^2^	0.9936	Std. Dev.	0.51				
Adj R^2^	0.9878	Mean	14.48				
Pred R^2^	0.9774	C.V.%	3.49				
Adeq Precision	40.40	PRESS	8.98				

The independent factors are: X_1_ (pH); X_2_ (peptone; g/L); X_3_ (Cantaloupe juice; g/L).

*Significant values, *F*: Fishers's function, *P*: Level of significance, C.V: Coefficient of variation.

The negative coefficient values suggest that the variables negatively affect the bacterial nanocellulose production by *Bacillus* sp*.* strain SEE-3, whilst the positive coefficient values imply a synergistic interaction among the factors and contribute to the improvement of the bacterial nanocellulose production by *Bacillus* sp*.* strain SEE-3. The model terms are significant as indicated by the Fisher’s *F* test (*F-*value = 171.31) with a very low *P* value (< 0.0001) and a lack of fit that is not statistically significant (*P* value = 0.5775) (Table 5). Furthermore, the coefficient of variation, PRESS (residual sum of squares), the adequate precision and standard deviation values were 3.94%, 8.98, 40.40 and 0.51; respectively. Furthermore, it is obvious from the *P* values of the coefficients that all linear coefficients, quadratic effects of X_1_, X_2_ and X_3_, as well as all interactions between the three factors tested (X_1_ X_2_, X_1_ X_3_ and X_2_ X_3_) are significant and affect the bacterial nanocellulose production by *Bacillus* sp*.* strain SEE-3 (Table 5).

The fit summary results (Supplementary Table S3) contributed to select the proper model that fit the bacterial nanocellulose production by *Bacillus* sp*.* strain SEE-3. The quadratic model is a highly significant and sufficient model for nanocellulose biosynthesis with a very low probability value (*P* value > 0.0001) and non-significant lack of fit (*F*-value 0.83, *P* value 0.5775). The quadratic model summary statistics had the best adjusted (0.9878) and predicted R^2^ (0.9774) values, as well as the lowest standard deviation (0.51).

The regression coefficients were calculated and then fitted into a polynomial equation of the second order to determine the relationship between various parameters and to calculate the greatest nanocellulose production according to the appropriate pH, peptone concentration, and Cantaloupe juice concentration. The bacterial nanocellulose production (Y) by *Bacillus* sp*.* strain SEE-3 can be predicted by the following regression equation:

5 $$\begin{aligned} {\text{Y}} & = + 19.01 + 0.58{\text{X}}_{1} + 1.23{\text{X}}_{2} + 2.06{\text{X}}_{3} - 1.21{\text{ X}}_{1} {\text{X}}_{2} - 1.08{\text{X}}_{1} {\text{X}}_{3} \\ & \quad + 0.76{\text{X}}_{2} {\text{X}}_{3} - 1.72{\text{ X}}_{1}^{2} - 2.89X_{2}^{2} - 4.45X_{3}^{2} \\ \end{aligned}$$

where Y was the predicted bacterial nanocellulose production and the coded levels of independent factors were: X_1_ (pH); X_2_ (peptone concentration; g/L); X_3_ (Cantaloupe juice; %, v/v).

### Three-dimensional surface and contour plots

**Figure 8:**
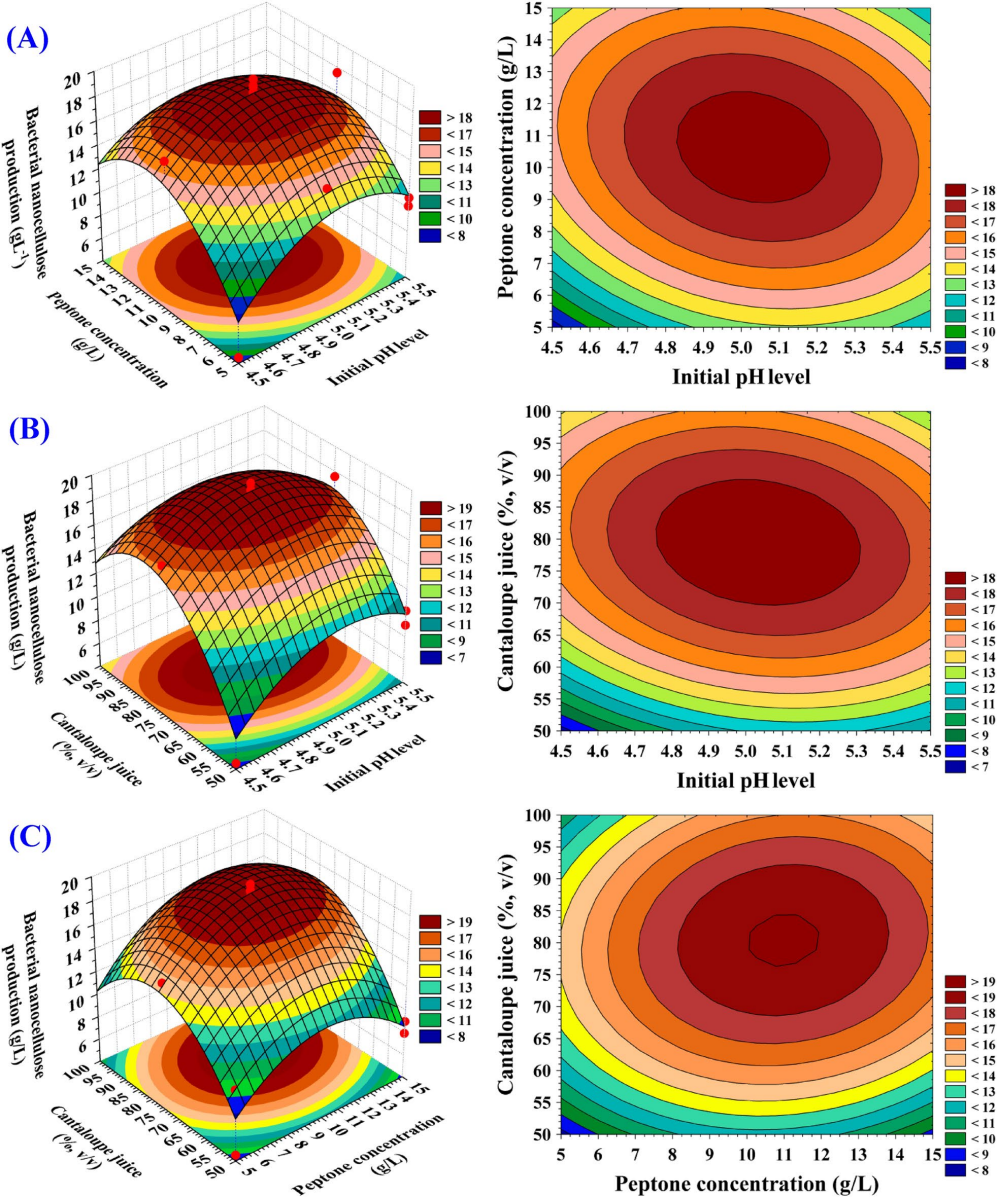
(**A**–**C)** 3D plots showing the mutual effects of pH (X_1_), peptone concentration (X_2_) and Cantaloupe juice (X_3_) on the bacterial nanocellulose production. This figure was created by using statistical software package, STATISTICA software (Version 8.0, StatSoft Inc., Tulsa, USA).

The three-dimensional surface plots (Fig. 8A–C) were created to determine the best levels and interactions between the variables: X_1_ (pH); X_2_ (peptone; g/L) and X_3_ (Cantaloupe juice; g/L) in order to identify the optimal conditions for maximum nanocellulose production. The three-dimensional surface plots were generated for the pair-wise combinations of the three significant variables, (X_1_ X_2_, X_1_ X_3_ and X_2_ X_3_) by drawing the bacterial nanocellulose production on the Z-axis against X and Y-axes for two independent variables while fixing the value of the third variable at center point.

Figure 8A shows the bacterial nanocellulose production by *Bacillus* sp*.* strain SEE-3 as a function of initial pH (X_1_) and peptone concentration (X_2_) while the Cantaloupe juice (X_3_) is kept at zero level. The maximum nanocellulose production was achieved at the moderate levels of initial pH level and peptone concentration. On the other hand, a gradual decrease in the nanocellulose production was shown to be associated with the highest and lowest levels of both initial pH level and peptone concentration.

The pH of the medium strongly affects the bacterial nanocellulose production and that the optimal pH for the bacterial nanocellulose production is in the range of 4–6, as this is a favorable pH range for the bacteria^108^. Most studies have stated that there is a marked trend towards acidification, began with an acidic pH of 4.5–6, with a desired value of 5^19^. Previously, Chen et al.^108^ studied the effect of pH on the production of the bacterial nanocellulose. They reported that the culture medium with an initial pH of 4 was better for the bacterial nanocellulose production using submerged cultivation. The best production of the bacterial nanocellulose by the DHU-ATCC-1 strain was achieved in a medium with an initial pH of 4 via submerged cultivation^108^. Urbina et al.^109^ reported that a pH of less than 4 is not suitable for the bacterial growth. By contrast, Urbina et al.^110^ reported that *Gluconacetobacter medellensis* cell viability was favoured in low pH media. The optimum initial pH for the bacterial cellulose production by cellulose producing bacterial strain, *Gluconacetobacter* sp. gel_SEA623-2, was 3.5^57^. Also, Al-Abdallah and Dahman^111^ reported that a pH of 2.0 is considered to be a suitable pH when *G. xylinus* ATCC 700,178 and wheat straw are used as the growth medium. On the other hand, Kiziltas et al.^112^studied the pH effect on the bacterial nanocellulose production; they found that an alkaline pH 8 was the optimum pH for the cultivation of *A. xylinus* 23,769 at an incubation temperature of 28 °C in hot water extracted-wood.

Each strain that produces cellulose needs a unique complex nitrogen source that supplies not only amino acids but also vitamins and mineral salts as well. The most commonly used nitrogen sources applied in numerous studies for bacterial cellulose production are peptone and yeast extract, which are the fundamental building blocks of the model medium being developed by Hestrin and Schramm^35^. However, the CSL was used as nitrogen source for agitated cultures^113^.

Figure 8B shows the production of nanocellulose as a function of initial pH level (X_1_) and Cantaloupe juice concentration (X_3_) while peptone concentration (X_2_) is kept at zero level. It can be seen that, the bacterial nanocellulose production increased gradually as the value of the initial pH and Cantaloupe juice concentration increased. Maximum bacterial nanocellulose production achieved at moderate levels of both Cantaloupe juice and initial pH that decreased with further increase in the initial pH value or Cantaloupe juice concentration.

The bacterial nanocellulose is synthesised by *Gluconacetobacter* using a variety of carbon sources. Glucose is the most frequently employed substrate since it is both a source of energy and a suitable precursor for the production of cellulose. However, the yield of bacterial nanocellulose may be limited due to the presence of glucose dehydrogenase in the cell membrane of *G. xylinus*. This enzyme converts glucose to gluconic acid, lowering the pH of the culture and thus interfering with bacterial nanocellulose manufacturing^114^. Fruits are distinguished by a high concentration of carbohydrates such as fructose and glucose. They have a low pH (particularly juices and extracts), which enables them to be used to culture microorganisms such as the acetic bacteria that produce cellulose^115^. Kurosumi et al.^60^ reported that the maximum bacterial nanocellulose production with *Acetobacter xylinus* NBRC 13,693 was 5.9 and 4.1 g/L using a suitable fruit juice like orange juice and pineapple; respectively in HS medium. The maximum bacterial nanocellulose production with extract of pineapple peel waste was found as 11.4 g/L^116^. On the other hand, Güzel and Akpınar^95^ have reported that the highest bacterial nanocellulose production by *Komagataeibacter hansenii* GA2016 was 1.54% and 11.53% for apple peel and kiwifruit hydrolysates; respectively. The sugar composition values of Cantaloupe juice were (g/100 mL): sucrose 1.73 , glucose 1.23 and fructose 1.61^42^.

Figure 8C shows the bacterial nanocellulose production by *Bacillus* sp*.* strain SEE-3 as a function of peptone concentration (X_2_) and the Cantaloupe juice concentration (X_3_) while the initial pH value (X_1_) is kept at zero level. The maximum nanocellulose production was achieved at the moderate levels of peptone and the Cantaloupe juice concentrations. On the other hand, a gradual decrease in the nanocellulose production was shown to be associated with the highest and lowest levels of both peptone concentration and the Cantaloupe juice concentrations.

### Model adequacy checking

**Figure 9:**
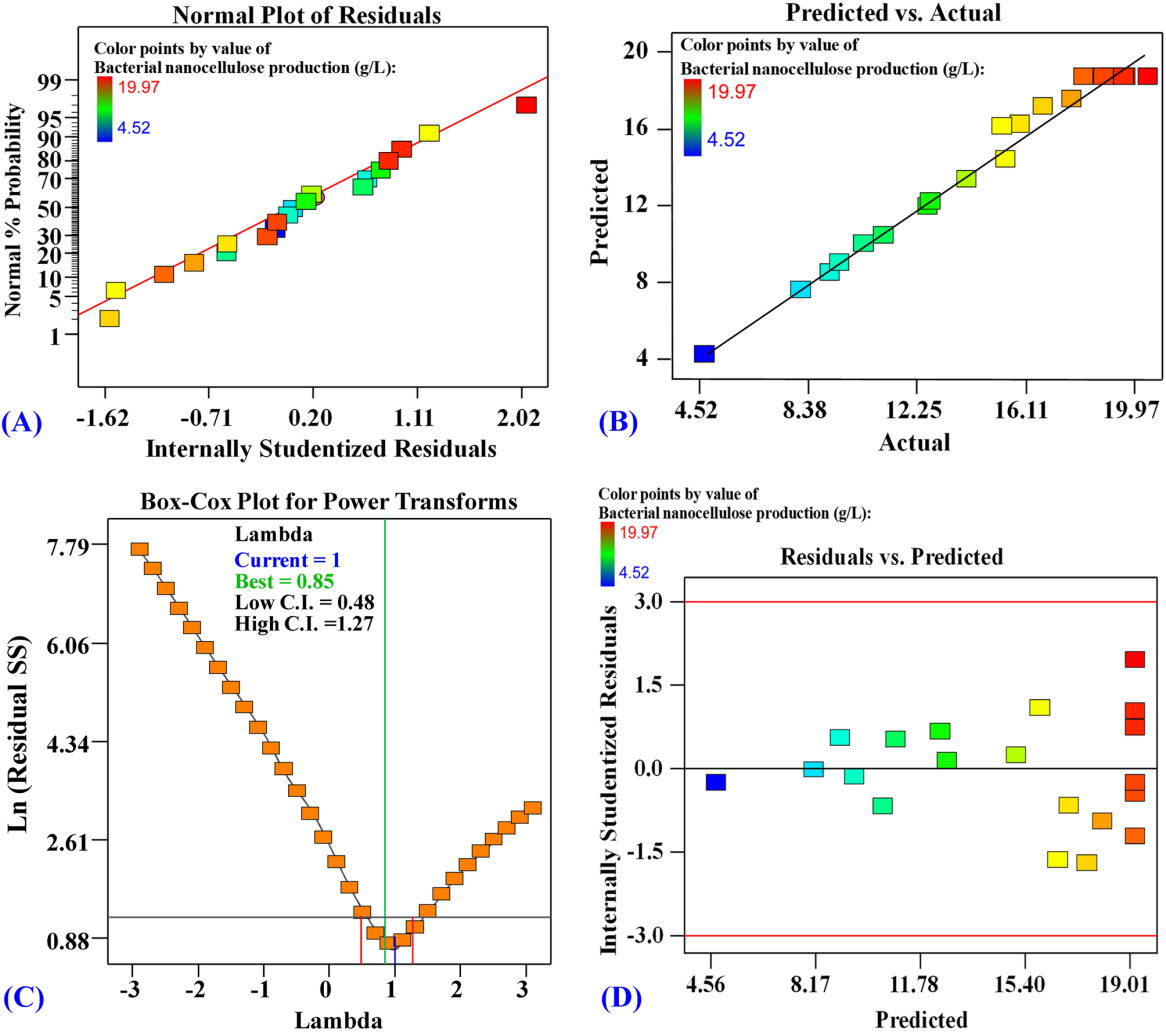
(**A**) Normal probability plot of internally studentized residuals, (**B**) plot of predicted versus actual, (**C**) Box–Cox plot of model transformation and (**D**) plot of internally studentized residuals versus predicted values of bacterial nanocellulose production. This figure was created by using Design Expert version 12 for Windows software.

The residuals' normal probability plot (NPP) is a crucial graphical tool for visualising the residuals' distribution and assessing the model's validity^117^. The residuals are the differences between the predicted and the experimental response values. Figure 9A shows the NPP of the studentized residuals. The residuals points are normal distributed; they are located adjacent to the diagonal line and shown in such a way that the points are regularly distributed, indicating the model's validity. Deviations from this straight line indicate that the residuals are not normally distributed. Figure 9B shows a plot of predicted versus actual values of the bacterial nanocellulose production, with points close to the fitted line, showing a significant correlation between the bacterial nanocellulose production values predicted by the model's and the experimental results, confirming the model's accuracy^118^. The Box–Cox graph of model transformation (Fig. 9C) shows the green line representing the best lambda value (Lambda (λ) = 0.85) and the blue line representing the current transformation optimal value (λ = 1). The red lines show the lowest and maximum values of confidence intervals between 0.48 and 1.27; respectively. The model is in the perfect zone and no data transformation is needed because of the blue line of the current transformation (λ = 1) fell between the minimum and maximum values of the confidence intervals (the two red lines; 0.48 and 1.27; respectively), indicating that the model fits the experimental data well. Figure 9D shows a plot of predicted bacterial nanocellulose production vs. studentized residuals. The residuals were distributed uniformly and randomly above and below the zero line, indicating a constant variance and demonstrating the model's accuracy.

### Desirability function (DF)

The  desirability function (DF) was used to define the optimal predicted conditions that would result in the greatest possible response. The values of DF ranged from 0 (undesirable) to 1 (desirable). The value of the desirability function is usually determined mathematically before the validation of the optimization process^119^. The Design Expert Software's (version 12) DF option was used to identify the optimal predicted conditions for the maximum response. Optimum bacterial nanocellulose production is illustrated by the optimization plot in Supplementary Fig. S1, which depicts the desirability function and predicted optimum values for maximum bacterial nanocellulose production. Using the optimised growth conditions, an experiment was carried out in triplicate to verify the bacterial nanocellulose production under the optimal predicted conditions. The obtained experimental bacterial nanocellulose (20.31 g/L) was then compared to the predicted bacterial nanocellulose production (19.42 g/L). The verification revealed that the experimental and predicted values of bacterial nanocellulose biosynthesis are in excellent agreement, implying that the DF successfully predicted the optimal values for highest bacterial nanocellulose production.

The optimal conditions for optimization experiments and a comparison between *Bacillus* sp*.* strain SEE-3 and some BC producers in terms of BC production and optimum conditions have been summarized in Table 6^116,120–127^.

**Table 6:** A comparison between *Bacillus* sp*.* strain SEE-3 and some BC producers in terms of BC production and optimum conditions.

Organism	Optimum conditions	BC production (g/L)	Reference
*Bacillus* sp*.* strain SEE-3	Medium volume; 100 mL/250 mL conical flask, initial pH level of 5, incubation time; 14 days, inoculum size; 5%, v/v, peptone; 11.22 g/L, citric acid; 1.5 g/L; Cantaloupe juice; 81.27%, v/v, yeast extract; 5 g/L, temperature; 37 °C, Na_2_HPO_4_; 3 g/L	19.42	Current study
*Komagataeibacter xylinus* IITR DKH20	70% (v/v) pineapple peel waste, citric acid conc. 1.15 g/L; peptone conc. 5 g/L; 2.7 g Na_2_HPO_4_ and yeast extract concentration was 5 g/L, at a temperature of 28 ± 2 °C, pH 6 after 16 days	11.4	Khan et al.^116^
*Komagataeibacter xylinus* ATCC 700,178	1.26 g/L citric acid and 3.39 g/L Na_2_HPO_4_, 1.5% m/v recycled paper sludge hydrolysate, 1.45% m/v yeast extract/peptone, 1% v/v ethanol, pH 5.5 after 15 days of fermentation	5.69	Soares da Silva et al.^120^
*Lactobacillus hilgardii* IITRKH159	g/L: 50 fructose, 5 yeast extract, 5 (NH_4_)_2_SO_4_, 3 KH_2_PO_4_, 0.05 MgSO_4_, pH 5.5 in a static condition at 28 ± 2 °C, after 16 days	7.23 ± 0.59	Khan et al.^121^
*Komagataeibacter xylinus* BPR 2001	% (m/v): citric acid 0.115, Na_2_HPO_4_ 0.27, (NH_4_)_2_SO_4_ 0.63, CSL 1.91, molasses 5.38 and ethanol 1.38% (v/v), pH 5.5 after 9 days at 30 °C under static conditions	7.5 ± 0.54	Rodrigues et al.^122^
*Gluconacetobacter xylinus*	3 g/L tea, 20 g/L mannitol and 40 g/L corn steep liquor after 7 days at 30 °C	3.34	Nguyen et al.^123^
*B. licheniformis* strain ZBT2	HS medium supplemented with grape juice (5%, v/v), yeast extract (1.5% w/v) and peptone (1.5% w/v)	9.2	Bagewadi et al.^124^
*B. amyloliquefaciens* ZF-7	%: 5 glucose, 0.5 peptone, 0.5 yeast extract, 0.5 Na_2_HPO_4_,0.1 citric acid, 1.4 ethanol, pH 6.5 in culture media inoculated with 1% (v/v) cells suspension at 30 °C for 5 days under static conditions	6.2	Zhu et al.^125^
*Leifsonia* sp.	HS medium supplemented with maltose at 2% (w/v) and 5 mL/L of soy whey as nitrogen source, pH 6.5 at 30 °C after 7 days when the inoculum size is 10%	5.97	Rastogi and Banerjee^126^
*Rhodococcus* sp. MI 2	SH medium containing 1.5% sucrose, 0.9% yeast extract, 0.7% peptone and 5% (v/v) inoculum at pH 3.5 and 25 °C under static conditions after14 days	7.4	Tanskul et al.^127^

## Supplementary Information

Supplementary Information.

## Data Availability

The datasets generated during the current study are available in the National Center for Biotechnology Information (NCBI) repository, under the accession number of MN826326 (https://www.ncbi.nlm.nih.gov/nucleotide/MN826326.1?report=genbank&log$=nucltop&blast_rank=1&RID=MEKVRA6801R).
