# Supplementary material for: Bacterial nanocellulose production using Cantaloupe juice, statistical optimization and characterization
Source: Sci Rep. 2023 Jan 2;13:51. doi: 10.1038/s41598-022-26642-9 (PMC9807561; doi:10.1038/s41598-022-26642-9)
Supplement: Supplementary file 1 — Supplementary Information. [file 41598_2022_26642_MOESM1_ESM.pdf]

Original Article

Bacterial nanocellulose production using Cantaloupe juice, statistical optimization and characterization

[1]Noura El-Ahmady El-Naggar*, [2]A. B Abeer Mohammed, [2]Sahar E. El-Malkey

[1]Department of Bioprocess Development, Genetic Engineering and Biotechnology Research Institute, City of Scientific Research and Technological Applications (SRTA-City), Alexandria, 21934, Egypt.

[2]Microbial Biotechnology Department, Genetic Engineering and Biotechnology Research Institute, University of Sadat City, Egypt

Corresponding Author's information

Dr. Noura El-Ahmady Ali El-Naggar

Address:

Bioprocess Development Department,

Genetic Engineering and Biotechnology Research Institute,

City of Scientific Research and Technological Applications,

New Borg El- Arab City, 21934, Alexandria, Egypt

Tel: (002)01003738444

Fax: (002)03 4593423

E-mail: nouraalahmady@yahoo.com

Results

Supplementary Table S1. List of different isolated bacterial nanocellulose producing bacterial strains.

Isolate no.	Dry wt. of bacterial nanocellulose (g/L)
SEE-1	–
SEE-2	–
SEE-3	9.4
SEE-4	–
SEE-5	–
SEE-6	–
SEE-7	3.7
SEE-8	–
SEE-9	6.2
SEE-10	5.9

Supplementary Table S2. Biochemical tests and identification of *Bacillus amyloliquefaciens* strain SEE-3.

**Characteristics**	**Properties**
Shape of colony	Irregular
Margin	Undulate
Elevation	Flat
Shape of growth in LB	On surface
Shape	Rods
Flagella	Flagellated
Motility	+ve
Gram Staining	+ve
Pigment	–ve
Spore	+ve
H_2O_2 production	+ve
Casein hydrolysis	+ve
Gelatin hydrolysis	+ve
Growth in 6.5% NaCl	+ve
**Utilization of**	
Glucose	+ve
Glycine	+ve
Mannitol	+ve
Fructose	+ve
Sucrose	+ve
CMC	+ve
Starch	–ve
D-ribose	+ve
D-xylose	+ve
D-sorbitol	+ve
D-raffinose	+ve
D-galactose	–ve
Lactose	–ve
D-maltose	–ve
D-mannose	–ve
D-trehalose	–ve

Supplementary Table S3. Fit summary for the results of FCCCD of bacterial nanocellulose production by *Bacillus amyloliquefaciens* strain SEE-3.

**Model Summary Statistics**					
**Source**	**SD**	**R^2**	**Adjusted R^2**	**Predicted R^2**	**PRESS**
Linear	4.59	0.1526	-0.0063	-0.5376	610.46
2FI	4.89	0.2174	-0.1438	-4.7412	2279.32
Quadratic	0.51	0.9936	0.9878	0.9774	8.98
**Lack of Fit Tests**					
**Source**	***SS***	***Df***	***MS***	***F*-value**	***P*-value**
Linear	335.02	11	30.46	109.05	< 0.0001
2FI	309.29	8	38.66	138.43	< 0.0001
Quadratic	1.16	5	0.23	0.83	0.5775
**Fit Summary**					
**Source**	**Sequential *P*-value**	**Lack of Fit *P*-value**	**Adjusted R^2**	**Predicted R^2**	
Linear	0.4353	< 0.0001	-0.0063	-0.5376	
2FI	0.7837	< 0.0001	-0.1438	-4.7412	
Quadratic	< 0.0001	0.5775	0.9878	0.9774	Suggested

* Significant values, *df*: degree of freedom, PRESS: sum of squares of prediction error, two factors interaction: 2FI

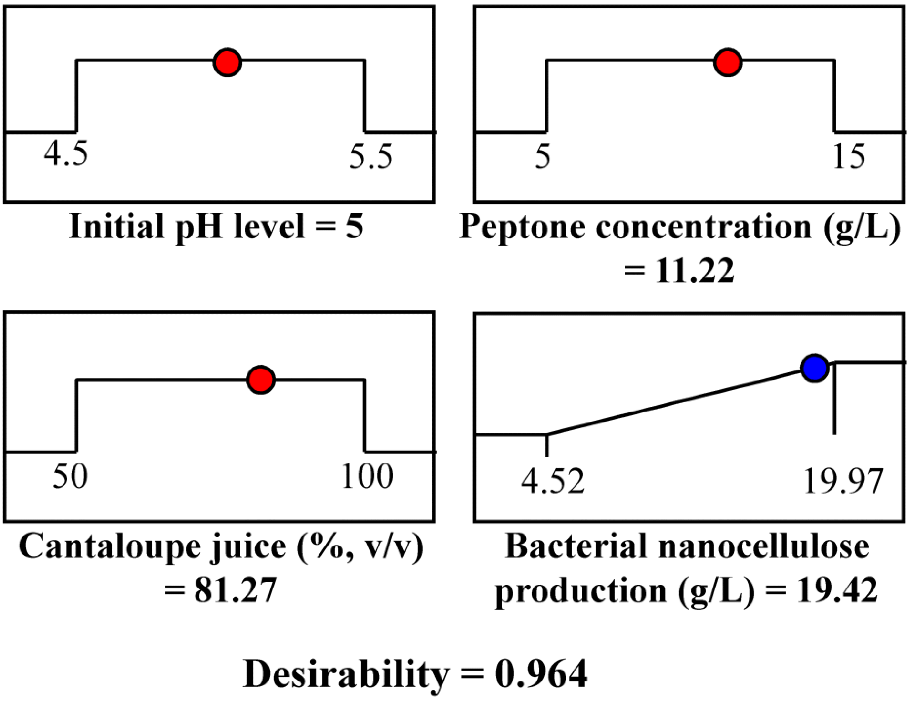

Supplementary Figure S1. The optimization plot displays the optimum predicted values for e maximum bacterial nanocellulose production by *Bacillus amyloliquefaciens* strain SEE-3 and the desirability value.

Complete gel for Figure 4 A

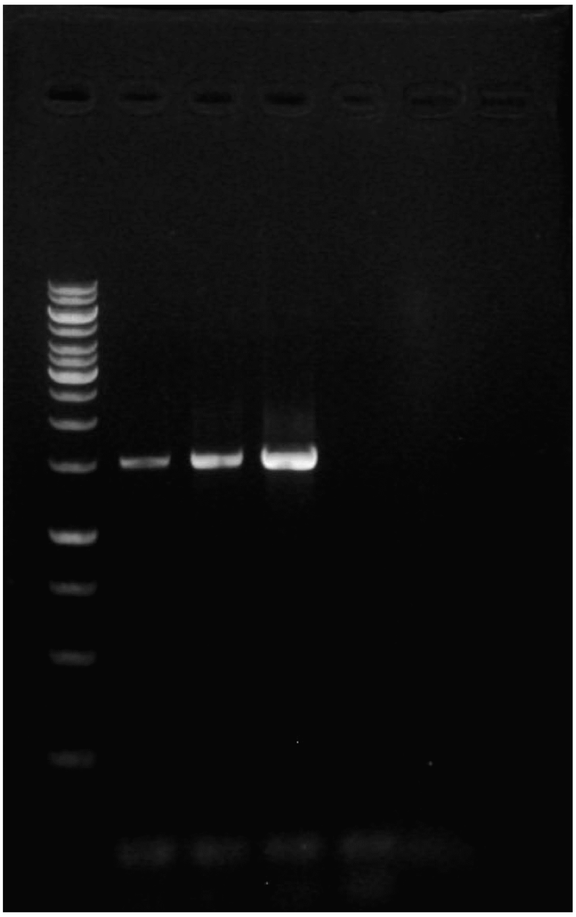

Uncropped image of agarose gel of PCR product bands of the amplified 16S fragment.
